# NAT10 inhibition corrects nuclear defects in tau mutant human neurons and extends lifespan in a *Drosophila* tauopathy model

**DOI:** 10.1016/j.isci.2026.116861

**Published:** 2026-07-22

**Authors:** Francesco Paonessa, Bernardo Delarue Bizzini, Tom Campbell, Emily Coode, Jonathan Lam, Ravi Solanki, Richard Butler, James Smith, Catherine M. Davidson, Delphine Larrieu, Andrea H. Brand, Frederick J. Livesey

**Affiliations:** 1 Zayed Centre for Rare Disease, UCL Great Ormond Street Institute of Child Health, University College London, London, UK; 2 Talisman Therapeutics, Babraham Research Campus, Cambridge CB22 3AT, UK; 3 The Gurdon Institute, University of Cambridge, Cambridge CB2 1QN, UK; 4 Department of Physiology, Development and Neuroscience, University of Cambridge, Cambridge CB2 3DY, UK; 5 Department of Cell Biology, NYU Grossman School of Medicine, New York, NY 10016, USA; 6 Cambridge Institute for Medical Research, University of Cambridge, Cambridge CB2 0XY, UK; 7 Department of Pharmacology, University of Cambridge, Cambridge CB2 1PD, UK

**Keywords:** Alzheimer’s, dementia, tau protein, human iPSCs, MAPT, *Drosophila*, neurodegeneration

## Abstract

Mutations in the gene encoding the microtubule-associated protein tau (*MAPT*) that are causal for frontotemporal dementia result in nuclear envelope deformation and disrupted nucleocytoplasmic transport when expressed in human neurons. A small-molecule inhibitor of the acetyltransferase NAT10 has been shown to correct similar nuclear membrane defects in Hutchinson-Gilford progeria syndrome, primarily by modulating microtubule dynamics. We report here that NAT10 inhibition and loss of function correct nuclear membrane abnormalities in human *MAPT*-mutant neurons. Similarly, NAT10 inhibition and haploinsufficiency correct neuronal nuclear shape defects and extend lifespan *in vivo* in a *Drosophila* model of tauopathy. NAT10 inhibition changes microtubule dynamics and corrects aberrant nucleocytoplasmic transport, and NAT10 directly interacts with regulators of microtubule dynamics in human *MAPT*-mutant neurons. We conclude that NAT10 mediates neuronal pathologies in tauopathies and is a potential therapeutic target in these diseases.

## Highlights

• Nuclear membrane dysfunction is caused by tau protein in dementia
• Inhibition of NAT10 corrects tau-driven pathologies in human neurons
• Loss of NAT10 rescues tau-driven pathologies and extends lifespan in *Drosophila*
• NAT10 mediates pathological microtubules via known cytoskeleton regulators

## Graphical abstract

**Figure:**
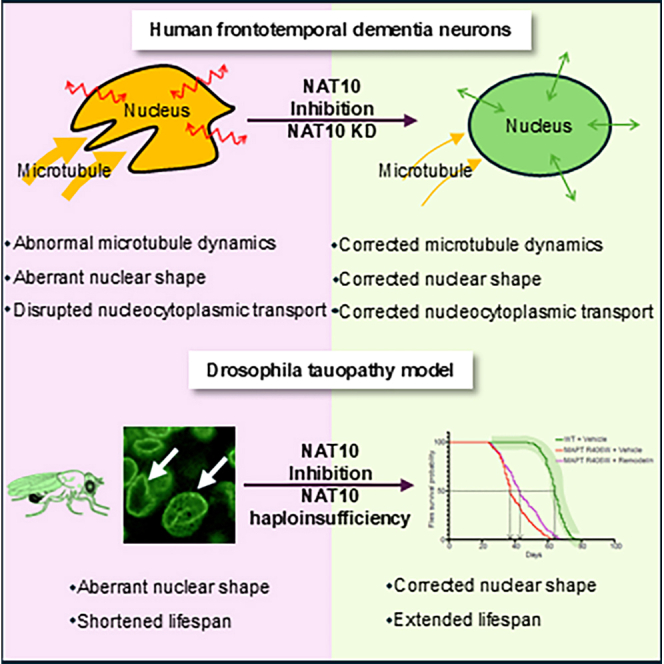

## Teaser

Biological sciences

## Introduction

The microtubule-associated protein tau (MAPT) is central to the pathogenesis of many neurodegenerative diseases, forming intracellular aggregates or neurofibrillary tangles in diseases collectively referred to as tauopathies, which include Alzheimer’s disease (AD), progressive nuclear palsy, and frontotemporal dementia (FTD).^1^^,^^2^ Autosomal dominant mutations in the gene encoding MAPT are causal for a highly penetrant form of FTD (FTLD-tau), with more than 50 pathogenic missense and intronic *MAPT* mutations identified to date.^1^^,^^3^ The majority of missense mutations are in and around the microtubule-binding region, promoting protein aggregation, whereas the intronic mutations alter splicing of the repeat-encoding exon 10, increasing the amount of the 4-microtubule binding repeat (4R) form of tau, relative to the 3-repeat (3R) form.^1^

Previously, we reported that both splicing (*MAPT* IVS10 + 16) and missense (*MAPT* P301L) *MAPT* mutations result in structural abnormalities of the nuclear membrane in human neurons *in vitro* and *in vivo*, resulting in dysfunctional nucleocytoplasmic transport *in vitro.*^4^ Similar tau-associated nuclear shape abnormalities have been reported in Huntington’s disease^5^ and AD.^6^ Tau-mediated disruption of the nuclear pore complex has been proposed as a pathogenic mechanism altering nucleocytoplasmic transport in AD.^7^ Notably, *Drosophila* models of tauopathy also exhibit nuclear abnormalities, with accumulation of mRNA in and around nuclear invaginations,^8^^,^^9^ together with disrupted heterochromatin.^6^

The neuronal nuclear abnormalities observed in AD and FTD are similar to those identified in Hutchinson-Gilford progeria syndrome (HGPS), a rare inherited laminopathy characterized by accelerated aging of non-neural tissues.^10^ HGPS results from a mutation in the *LMNA* gene that impairs processing of prelamin A protein.^10^ This leads to the accumulation of a shorter, toxic form of lamin A, known as progerin, which disrupts nuclear membrane integrity with a characteristic cellular phenotype of nuclear blebbing. In cellular models of HGPS, there is a disruption of cytoskeletal and microtubule dynamics as well as alterations in nucleocytoplasmic transport.^11^^,^^12^^,^^13^ We (D.L.) previously found that the small molecule Remodelin, an inhibitor of the NAT10 acetyltransferase, corrects both nuclear shape abnormalities and the disruption of nucleocytoplasmic transport in HGPS cells.^13^ Furthermore, both Remodelin treatment and haploinsufficiency of NAT10 increased health and lifespan in a mouse model of HGPS.^11^

Given the similarities in the cellular phenotypes in human FTD-MAPT neurons and HGPS fibroblasts, we investigated the effects of inhibition or loss of function of NAT10 in human tauopathy models *in vitro* and in a *Drosophila* tauopathy model *in vivo*. We report that inhibition of NAT10 corrected nuclear membrane deformation in human iPSC-derived FTD-MAPT neurons. Small interfering RNA (siRNA)-mediated knockdown (KD) of NAT10 also corrected nuclear defects in FTD neurons, confirming that inhibition or loss of function of NAT10 rescues this phenotype in human neurons. In a *Drosophila* tauopathy model, inhibition of dNAT10 or haploinsufficiency of dNAT10 corrected nuclear membrane abnormalities and significantly extended animal lifespan. NAT10 inhibition reduced microtubule elongation and mechanical stress on the nuclear envelope in FTD neurons, improving nucleocytoplasmic transport. Immunoprecipitation-mass spectrometry (IP-MS) analysis of NAT10 in human control and FTD neurons identified several regulators of microtubule dynamics as NAT10-interacting proteins, confirmed by additional co-immunoprecipitation (coIP) studies. These results indicate that NAT10 mediates microtubule-mediated nuclear pathologies in FTD neurons and represents a potential therapeutic target for tauopathies.

## Results

### A small-molecule inhibitor of the NAT10 acetyltransferase corrects nuclear lamina defects in human FDT-MAPT neurons

We previously reported that pathogenic mutations in the *MAPT* gene resulted in abnormal microtubule dynamics in the neuronal cell body, deforming the nuclear membrane and disrupting nucleocytoplasmic transport.^4^ These phenotypes are very similar to those observed in non-neural cells in the premature aging condition, HGPS, which are corrected by a small-molecule inhibitor of the NAT10 acetyltransferase, Remodelin.^13^ Therefore, we investigated whether nuclear lamina defects in human FTD-MAPT neurons are affected by NAT10 inhibition.

To do so, cortical excitatory neurons were generated from iPSCs derived from two different individuals carrying the pathological *MAPT* IVS10 + 16 mutation (*MAPT* patient A and *MAPT* patient B); an isogenic revertant induced pluripotent stem cell (iPSC) line derived from the patient B line in which the mutation was corrected by genome editing (*MAPT* control); and an unrelated, independent non-demented control (NDC) line (Control). One hundred twenty (120) days from the initiation of neuronal differentiation, neurons were treated with the NAT10 inhibitor Remodelin for 48 h and the frequency of nuclear lamina invaginations in neurons was scored based on lamin B1 immunostaining and image analysis (see STAR Methods for details).

**Figure 1:**
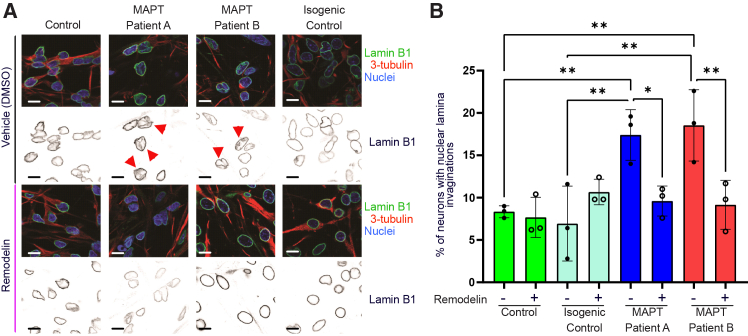
The NAT10 small-molecule inhibitor Remodelin corrects nuclear lamina defects in human FTD-MAPT neurons (A) Confocal images of the nuclear lamina (lamin B1, green or gray) in FTD-MAPT neurons (MAPT patient A and MAPT patient B) compared with control and MAPT IVS10 + 16-B isogenic revertant (MAPT Control) neurons (120 days-in-vitro (DIV); β3-tubulin, red; DAPI, blue). Neurons were exposed to 7.5 μM Remodelin or vehicle control (DMSO) for 48 h. Abnormalities of nuclear lamina shape are common in FTD-MAPT neurons, while nuclear lamina invaginations are reduced by inhibition of NAT10 with Remodelin. Scale bars, 10 μm. (B) FTD-MAPT neurons had an increased incidence of neurons with nuclear lamina invaginations compared with control neurons in vehicle (−), which was significantly reduced after treatment with Remodelin (+). Significance was determined by two-way ANOVA followed by Holm-Sidak’s multiple comparison test (∗*p* < 0.05; ∗∗*p* < 0.01); data are represented as mean ± SD; *n* = 3 independent experiments, each dot represents the average of one experiment, with *n* = 3 wells per experiment.

Consistent with our previous data, FTD-MAPT neurons had a higher frequency of nuclear lamina invaginations compared to control neurons (Figures 1A and 1B). Remodelin treatment reversed this phenotype, reducing the proportion of neurons with nuclear lamina defects to control levels (Figures 1A and 1B). Remodelin treatment had minor effects on lamin B1 and A/C protein levels in control and FTD-MAPT neurons (Figures S1A and S1B). To confirm that the correction in nuclear lamina shape following Remodelin treatment measured by lamin B immunostaining reflected changes in nuclear membrane morphology, independent of lamin B protein levels, nuclear shape was also measured using DAPI-based nuclear solidity (Figure S1C). This analysis confirmed that nuclear shape is distorted in FTD-MAPT neurons and corrected by Remodelin treatment (Figures S1C and S1D).

### Small-molecule inhibition of NAT10 corrects neuronal nuclear defects and extends survival in a *Drosophila* tauopathy model

*Drosophila melanogaster* is a well-established system for *in vivo* modeling of neurodegenerative diseases, including tauopathies.^14^ Transgenic expression of human *MAPT* variants with missense FTD mutations captures several features of disease, including neuronal degeneration, abnormal movement, and reduced lifespan.^6^^,^^9^^,^^15^ Disruption of the nuclear lamina and chromatin reorganization have been reported in *Drosophila* expressing human *MAPT* R406W throughout the nervous system,^9^ and *Drosophila* expressing human *MAPT* R406W also has a shortened lifespan.^15^

**Figure 2:**
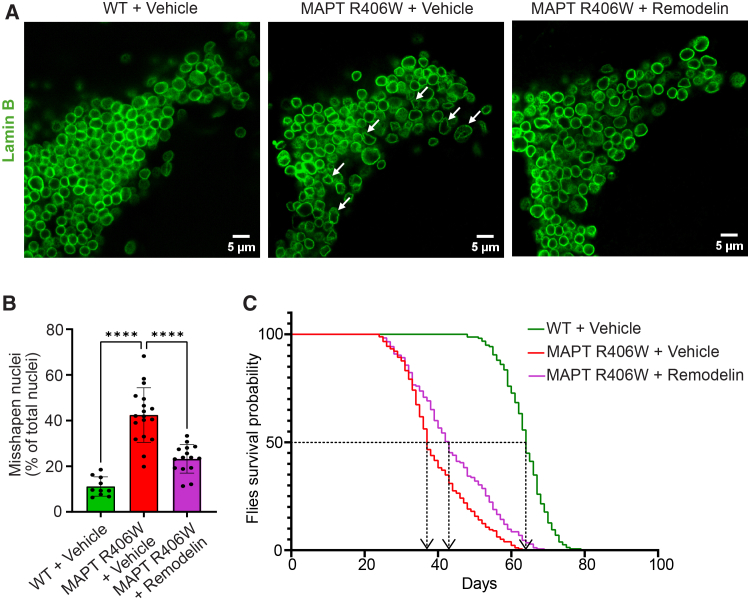
The NAT10 small-molecule inhibitor Remodelin corrects nuclear lamina morphology defects and extends lifespan in the *Drosophila* MAPT R406W tauopathy model (A) Confocal images of the nuclear lamina (lamin B, green) in neurons on the ventral side of the antennal lobe of 44-day-old control (WT; w1118; +; +) and elaV > MAPT R406W *Drosophila* (pan-neuronally expressing MAPT R406W), fed on either vehicle (20% DMSO, 65% 2-hydroxypropyl-b-cyclodextrin, and 15% Tween 80) or Remodelin (100 μM)-containing food throughout development and lifespan. Neurons in the antennal lobe displayed frequent nuclear lamina defects in vehicle-treated MAPT R406W (arrows), whereas neurons from animals exposed to Remodelin show improved nuclear morphology. Scale bars, 5 μm. (B) Quantification of the frequency of misshapen nuclei was performed on neurons from 44-day-old WT and elaV > MAPT R406W *Drosophila* neurons. MAPT R406W animals exposed to vehicle alone show an increased percentage of misshapen neuronal nuclei compared to WT flies. Remodelin treatment significatively reduces the frequency of misshapen nuclei in MAPT R406W animals. Significance was tested by two-way ANOVA followed by Tukey’s multiple comparison test (∗∗∗∗*p* <0.0001); data are represented as mean ± SD; WT + vehicle *n* = 10, R406W + vehicle *n* = 18 and R406W + Remodelin *n* = 15 representing one section per antennal lobe and hemisphere (max. 2 sections per animal). Dots represent the percentage of misshapen nuclei per brain area scored. (C) A lifespan experiment was performed to assess the effect of Remodelin on the longevity of *Drosophila* pan-neuronally expressing MAPT R406W. Flies expressing MAPT R406W showed a reduced lifespan compared with WT animals, with median survival decreased by 42% (37 vs. 64 days; hazard ratio [HR] = 15.65, 95% confidence interval [CI]: 11.16–21.94). Remodelin treatment throughout development and adult life increased median lifespan of MAPT R406W animals by 16%, compared to the vehicle-only MAPT R406W group (median lifespan 43 vs. 37 days; HR = 0.62, 95% CI: 0.50–0.77). Median survival (50% survival probability) is highlighted (dotted lines and arrows) for each group. Survival was recorded for *n* = 178 (MAPT R406W + Vehicle), *n* = 175 (MAPT R406W + Remodelin) and *n* = 158 (WT + Vehicle) female flies.

To determine whether inhibition of NAT10 with Remodelin corrects nuclear membrane defects *in vivo*, *MAPT* R406W-expressing animals were maintained on food containing either vehicle or 100 μM Remodelin, the highest concentration without developmental toxicity (Figure S2). As previously reported,^9^ pan-neuronal expression of *MAPT* R406W resulted in neuronal nuclear lamina invaginations in adult flies (Figures 2A and 2B). Remodelin treatment reduced the number of neuronal nuclei with nuclear membrane defects in the *Drosophila* tauopathy model (Figures 2A and 2B), consistent with the effect of NAT10 inhibition in human FTD-MAPT neurons.

Transgenic expression of both wild-type (WT) and mutant forms of human tau is associated with shortened lifespan in *Drosophila*.^16^ Therefore, we investigated whether NAT10 inhibition affected overall fitness in transgenic animals expressing human *MAPT* R406W. As previously reported, we found that survival was greatly reduced in animals expressing human *MAPT* R406W, with 50% survival probability reduced from approximately 64 to 37 days (Figure 2C). Remodelin treatment increased survival of *MAPT* R406W-expressing animals, with the 50% survival probability increased to 43 days (Figure 2C). Thus, small-molecule inhibition of NAT10 both prevented tau-mediated neuronal nuclear membrane defects *in vivo* and extended lifespan in animals expressing the pathogenic *MAPT* R406W variant.

### Reduction of NAT10 protein corrects nuclear lamina defects in human FTD-MAPT neurons

To confirm that the effects of Remodelin in human neurons are via its known target, NAT10, we sought to generate *NAT10* loss-of-function FTD neurons. Consistent with evidence that *NAT10* is an essential gene for development in mice,^11^ we could not successfully generate viable *NAT10* knockout (KO) human iPSCs by CRISPR-mediated gene targeting. Similarly, efforts to specifically target NAT10’s acetyltransferase domain to remove its enzymatic activity did not yield viable targeted iPSC clones (data not shown).

**Figure 3:**
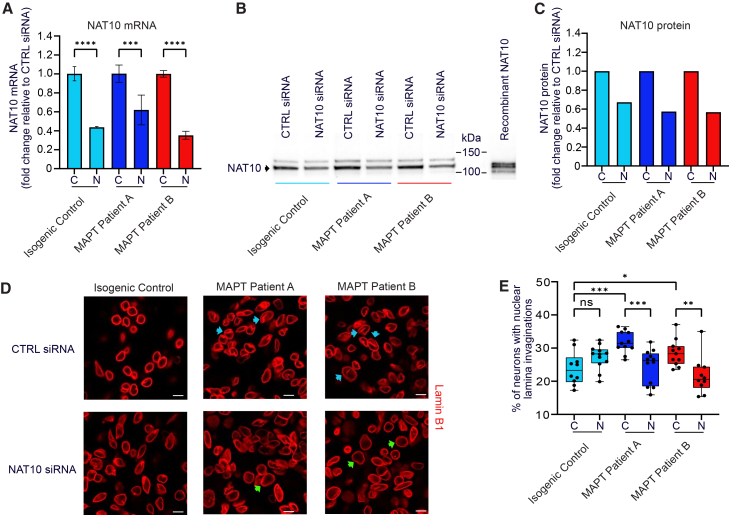
Acute reduction of NAT10 protein corrects nuclear lamina defects in human FTD-MAPT neurons (A) NAT10 siRNA treatment for 72 h reduces NAT10 mRNA by over 60% in control and FTD neurons, compared with scrambled siRNA treatment (C = CTRL siRNA; N = NAT10 siRNA). Significance was determined using two-way ANOVA followed by Fisher’s post hoc test (∗∗∗*p* < 0.001; ∗∗∗∗*p* < 0.0001); data are represented as mean ± SD. (B and C) Western blot (B) for NAT10 in neurons treated with CTRL siRNA or NAT10 siRNA. The NAT10 band was identified by size comparison with recombinant NAT10 protein. NAT10 siRNA reduces NAT10 protein by over 40% in control and FTD neurons, compared to scrambled siRNA treatment (C) (C = CTRL siRNA; N = NAT10 siRNA). (D and E) Reduction of NAT10 protein by siRNA KD significantly reduces the incidence of nuclear lamina defects in MAPT-FTD neurons, compared with control siRNA-treated neurons.(D) Representative confocal images from lamin B1 (red) in neurons treated with CTRL siRNA or NAT10 siRNA. Blue arrows indicate nuclei with nuclear lamina defects; green arrows indicate examples of normally shaped nuclei.(E) Percentages of neurons with lamin B1 invaginations are higher in FTD than control neurons, and reduced by NAT10 KD. Significance was determined using two-way ANOVA followed by Sidak’s multiple comparison test (ns, not significant, ∗*p* < 0.05; ∗∗*p* < 0.01, ∗∗∗*p* < 0.001); data are represented as box-and-whisker plot, boxes represent the median and interquartile range; whiskers indicate minimum and maximum values. Scale bars, 10 μm.

As an alternative strategy, we acutely knocked down NAT10 protein in neurons with siRNA (NAT10 KD). Following siRNA delivery, NAT10 mRNA was reduced by between 40% and 70% in neurons, with NAT10 protein reduced by 40%–50%, without impacting neuronal viability (Figures 3A–3C and S3). NAT10 KD corrected nuclear lamina defects in FTD-MAPT neurons compared to a scrambled control siRNA (Figures 3D and 3E): the increased proportion of FTD-MAPT neurons with nuclear lamina defects, compared to isogenic control neurons, was reduced by NAT10 KD to the level of healthy control neurons. Thus, both acute loss of NAT10 function and small-molecule inhibition of its acetyltransferase activity correct nuclear lamina defects in human FTD-MAPT neurons.

### *Drosophila* NAT10 haploinsufficiency corrects nuclear defects and extends lifespan in an *in vivo* tauopathy model

Given that the genetic KD of NAT10 corrects nuclear lamina defects in human FTD-MAPT neurons, we tested the effect of genetic loss of function of NAT10 in the *Drosophila MAPT*-R406W model. The *Drosophila* ortholog of NAT10, l(1)G0020 (dNAT10), shares 53% homology with human NAT10, and the acetyltransferase domain of dNAT10, the proposed target of Remodelin, is conserved between the species. The dNAT10 mutant line (l(1)G0020(1)/FM7c; +: +; Bloomington Drosophila Stock Center #11474) is homozygous lethal: only balanced males (FM7c/y) and heterozygous or homozygous balanced females (l(1)G0020/FM7c or FM7c/FM7c) emerge as adults. This is consistent with our results that *NAT10* KO iPSCs are not viable and with the previously reported lethality of homozygous *NAT10* mutant mice.^11^

**Figure 4:**
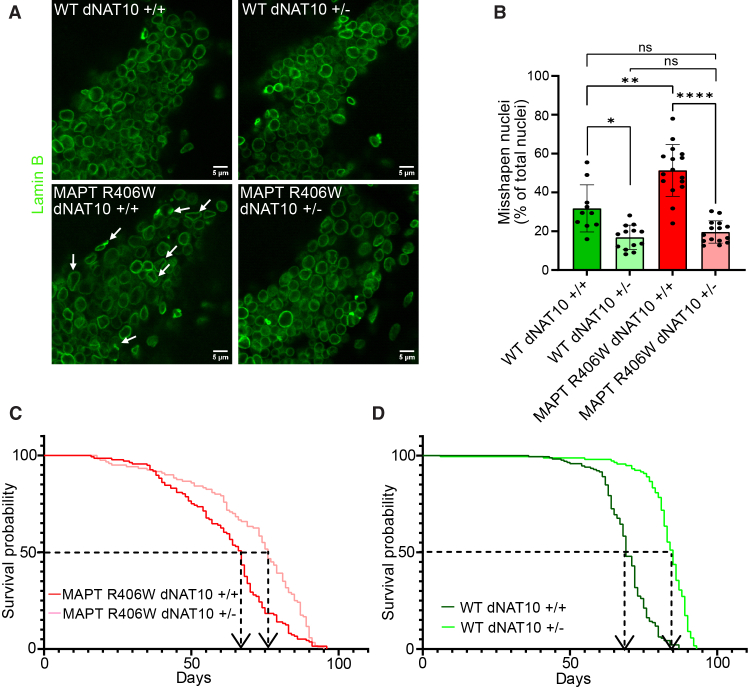
Haploinsufficiency of *Drosophila* NAT10 corrects nuclear lamina defects and extends lifespan in the MAPT R406W *Drosophila* tauopathy model (A) Confocal images of the nuclear lamina (lamin B, green) of antennal lobe neurons in 20-day-old WT (w1118) and elaV > R406W *Drosophila* neurons homozygous (dNAT10 +/+) or heterozygous (dNAT10 +/−) for NAT10. Neurons displayed frequent nuclear lamina defects in MAPT R406W flies (arrows), whereas R406W dNAT10 +/− mutant flies had fewer misshapen nuclei. Scale bars, 5 μm. (B) Quantification of misshapen nuclei was performed on 20-day-old WT or elaV > R406W *Drosophila* neurons. MAPT animals heterozygous for a mutant dNAT10 allele show a significantly reduced frequency of misshapen nuclei when compared to homozygous dNAT10 flies. Significance was tested using two-way ANOVA followed by Tukey’s multiple comparison test (ns, not significant, ∗*p* < 0.05; ∗∗*p* < 0.01, ∗∗∗∗*p* <0.0001); data are represented as mean ± SD; *n* = 10 WT dNAT10+/+, *n* = 13 WT dNAT10+/−, *n* = 17 R406W dNAT10+/+, and *n* = 16 R406W dNAT10+/− unique sections per brain hemisphere (max. 2 sections per animal); each dot represents the percentage of misshapen nuclei per brain section scored. (C and D) A longevity assay was performed to assess whether one copy of a mutant dNAT10 allele impacts lifespan in wild type or R406W tauopathy model. dNAT10 (±) animals showed a significantly improved median lifespan of 23% and 13% (85 vs. 69 days (wild type) and 76 vs. 67 days (R406W); HR = 0.12 and 0.59, 95% CI: 0.09–0.16 and 0.46–0.76, respectively; log rank *p* < 0.001 for both comparisons). Survival was recorded for *n* = 123 WT dNAT10+/+ and *n* = 159 WT dNAT10+/− (C) and *n* = 135 R406W dNAT10+/+ and *n* = 120 R406WdNAT10^+/−^ female flies.

Therefore, to investigate the effects of reducing NAT10 function in the *Drosophila* tauopathy model, we generated heterozygous *dNAT10* mutants expressing R406W *MAPT* (tau). As observed after Remodelin treatment, *dNAT10* heterozygous null animals expressing *MAPT* R406W had fewer neurons with nuclear lamina deformation than *MAPT*-R406W animals (Figures 4A and 4B). Furthermore, *MAPT*-R406W; heterozygous *dNAT10* mutant animals had increased lifespan compared with *MAPT*-R406W alone, increasing the 50% survival probability age from 67 to 76 days (Figure 4C). Notably, *dNAT10* haploinsufficiency also significantly reduced nuclear membrane misfolding and increased lifespan in WT flies (50% survival probability age from 69 to 85 days; Figure 4D), suggesting that NAT10 has a wider role in neuronal aging and longevity. Overall, reducing the activity of NAT10 *in vivo* corrects neuronal nuclear lamina defects and extends lifespan in a *Drosophila* model, consistent with the findings in human FTD-MAPT neurons.

### Nucleocytoplasmic transport defects in FTD-MAPT neurons are reversed by inhibition of NAT10

Impaired nuclear membrane integrity and disrupted nucleocytoplasmic transport are significant pathologies in several neurodegenerative diseases, including ALS and ALS-FTD.^17^ Disruption of nuclear pore complexes and aberrant nucleocytoplasmic transport have been found in ALS due to *C9ORF72* expansion,^18^ in AD^7^ and FTD-MAPT.^4^ In the latter study, we found that the aberrant nucleocytoplasmic transport in human FTD-MAPT neurons was corrected by acute exposure to the microtubule depolymerizing small-molecule nocodazole.^4^

**Figure 5:**
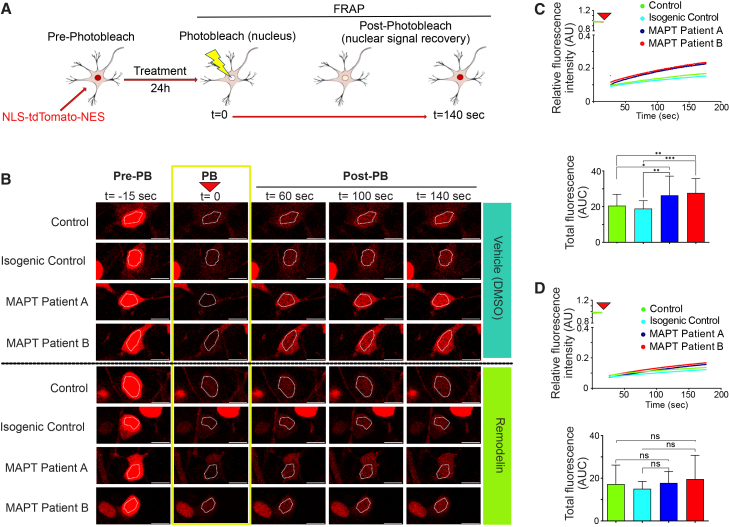
Nucleocytoplasmic transport defects in FTD-MAPT neurons are reversed by inhibition of NAT10 (A) Fluorescence recovery after photo bleaching (FRAP) workflow for neurons transduced with lentivirus expressing the NLS-tdTomato-NES reporter. Transduced control and FTD-MAPT neurons (120 DIV) were treated with DMSO or Remodelin for 24 h and then subjected to FRAP in the cell nucleus. (B) Representative images of neurons treated with DMSO or Remodelin before photobleaching of nuclear tdTomato (pre-PB), at t = 0 (PB), and t = 60, 100 and 140 s post-photobleaching (post-PB). The area exposed to photobleaching is highlighted with the white dotted line. Scale bars,10 μm. (C) FTD-MAPT neurons show a quicker recovery of nuclear FRAP of tdTomato relative to non-demented control neurons (all neurons treated with DMSO vehicle). FTD-MAPT neurons have statistically significantly more total nuclear FRAP (Area Under the Curve, AUC) compared to non-demented control neurons when treated with vehicle. Significance was determined using one-way ANOVA followed by Tukey’s test (∗*p* < 0.05; ∗∗*p* < 0.01; ∗∗∗*p* < 0.001); data are represented as mean ± SD; *n* > 15 movies from 3 independent experiments. (D) Following treatment with Remodelin, nuclear tdTomato shows a similar recovery rate after photobleaching between control and FTD-MAPT neurons. Curves represent the average of >15 movies. Following Remodelin treatment, nuclear FRAP in FTD-MAPT neurons is indistinguishable from that of non-demented controls. Significance was determined using one-way ANOVA followed by Tukey’s test (ns, not significant); data are represented as mean ± SD; *n* > 15 movies from 3 independent experiments.

We tested whether the correction of nuclear lamina defects by NAT10 inhibition was accompanied by an improvement in nucleocytoplasmic transport. To do so, we carried out fluorescent recovery after photobleaching (FRAP) experiments in iPSC-derived neurons expressing Shuttle-tdTomato, an NLS-NES-tagged TdTomato^18^ that dynamically moves between the nucleus and cytoplasm (Figure 5A). As expected, Shuttle-tdTomato was enriched in the nucleus when expressed in human iPSC-derived neurons, with less signal in the cytoplasm and neurites (Figure 5B; pre-PB condition).

Neurons expressing Shuttle-tdTomato were treated with vehicle (DMSO) or Remodelin for 24 h before FRAP analysis in neuronal nuclei. Note that the FRAP process resulted in a reduction in fluorescence beyond the targeted region, which is expected and reflects both optical and biological factors inherent to FRAP measurements. PB in confocal microscopy follows the point-spread function of the laser, resulting in a Gaussian-like bleaching profile. Consequently, fluorescence is partially reduced in a domain extending beyond the defined target area. In addition, diffusion also results in reduced fluorescence beyond the photobleached area, as bleached molecules and unbleached molecules diffuse between the region of interest (ROI) and the surrounding region during the imaging period.

Vehicle-treated FTD-MAPT neurons showed a faster recovery of nuclear tdTomato signal compared to either NDC or *MAPT* IVS10 + 16B isogenic control neurons, confirming aberrant nucleocytoplasmic transport (Figures 5B and 5C), consistent with our previous study.^4^ Inhibition of NAT10 by treatment with Remodelin for 24 h slowed the recovery of nuclear tdTomato in FTD-MAPT neurons, such that the rate of FRAP was indistinguishable between NAT10-treated FTD-MAPT, isogenic, and unrelated NDC neurons (Figures 5B and 5D). Therefore, NAT10 inhibition with Remodelin corrects nucleocytoplasmic transport defects in MAPT-FTD neurons.

### NAT10 inhibition alters microtubule dynamics in human FTD-MAPT neurons

Deformation of nuclear membranes in FTD-MAPT neurons is associated with altered microtubule dynamics within the neuronal cell body, with microtubules deforming the nucleus.^4^ Acute treatment with the microtubule-destabilizing compound, nocodazole, restored nuclear shape, demonstrating that nuclear membrane deformation is a dynamic process.^4^ Therefore, we investigated whether NAT10 inhibition with Remodelin impacts microtubule dynamics in both control and FTD-MAPT neurons.

**Figure 6:**
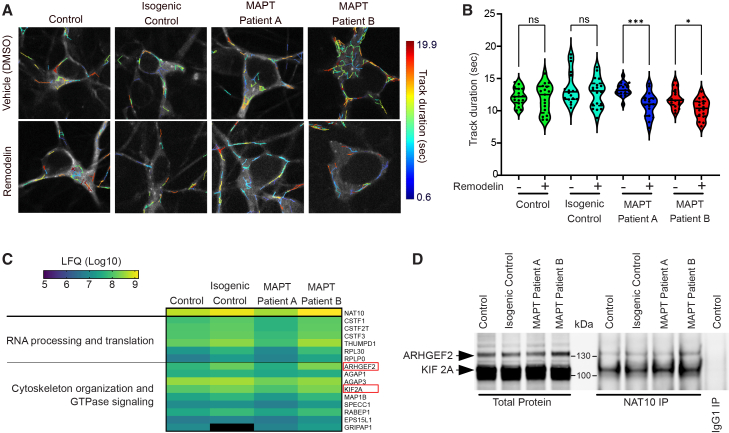
NAT10 inhibition alters microtubule dynamics in human FTD-MAPT neurons (A) Representative images of microtubule track duration (cumulative over a 150 s movie) visualized by live-imaging of an EB3-GFP fusion protein. EB-3 tracks are shown overlaid on movie stills of control and FTD-MAPT neurons (120 DIV) after 24 h treatment with vehicle (DMSO) or Remodelin. (B) Microtubule growth, reflected in EB3 track duration, is reduced by Remodelin treatment in FTD-MAPT neurons but not in control neurons (–= Vehicle; + = Remodelin). Significance was determined using two-way ANOVA followed by Sidak’s multiple comparison test (ns, not significant; ∗*p* < 0.05; ∗∗∗*p* < 0.001); data are represented as median with minimum and maximum values; *n* > 15 movies from 3 independent experiments, dots represent the average of one movie. (C) IP-MS of NAT10-interacting proteins in human FTD-MAPT neurons. Graphical representation of the label-free quantification (LFQ) counts for proteins identified by mass spectrometry following IP of NAT10 from neurons of each genotype listed (full list of identified proteins in Table S1). In addition to NAT10 itself, identified interactors are listed on the right and grouped based on Gene Ontology. ARHGEF2 and KI2A are highlighted in red boxes. (D) Confirmation of interactions between NAT10 and selected cytoskeleton regulatory proteins, ARHGEF2 and KIF2A. Immunoblotting for ARHGEF2 and KIF2A following IP of NAT10 from proteins extracts from FTD-MAPT or control neurons (NAT10 IP) demonstrated that both proteins were present, with no signal in protein extracts from control neurons subjected to coIP using rabbit IgG1 control. ARHGEF2 and KIF2A protein intracellular abundance is also shown for reference (total protein).

To do so, we introduced a construct driving expression of the microtubule tip-binding protein, EB3, fused to GFP and driven by the neuron-specific promoter, Synapsin1 (*pSyn1*:EB3-GFP) into control and FTD-MAPT human neurons (Figure 6). Neuronal expression of GFP was observed within 1 day, and most cells were positive within 3 days. To measure microtubule dynamics, single-plane videos of EB3-GFP were captured, enabling microtubule growth to be tracked and analyzed in individual neurons (Figure 6A; Videos S1, S2, S3, and S4).

Video S1. Examples of live imaging of EB3-GFP in control neurons treated with vehicle (DMSO) or Remodelin, related to Figure 6

Video S2. Examples of live imaging of EB3-GFP in isogenic control neurons treated with vehicle (DMSO) or Remodelin, related to Figure 6

Video S3. Examples of live imaging of EB3-GFP in MAPT IVS10 + 16 (patient A) neurons treated with vehicle (DMSO) or Remodelin, related to Figure 6

Video S4. Examples of live imaging of EB3-GFP in MAPT IVS10 + 16 (patient B) neurons treated with vehicle (DMSO) or Remodelin, related to Figure 6

Analyzing microtubule dynamics, we found that Remodelin treatment for 24 h shortened microtubule growth, as reflected in a reduction in EB3-GFP track duration (Figure 6B). However, Remodelin had no effect on microtubule growth (EB3 track duration) in isogenic and control neurons (Figure 6B). This decrease in microtubule elongation, which accompanies the correction of nuclear lamina defects, is consistent with our previous observation that nocodazole treatment rectifies nuclear lamina defects.^4^

To further explore how NAT10 inhibition alters microtubule dynamics to correct nuclear membrane shape, levels of total and phosphorylated tau protein, α-tubulin, acetylated tubulin, and β-actin were measured by immunoblotting (Figure S4). No significant changes in total levels of each protein were detected. Similarly, confocal imaging of acetylated microtubules and of actin filaments did not identify large-scale changes in the organization of the neuronal cytoskeleton after 48 h of Remodelin treatment (Figure S5).

Finally, to investigate in an unbiased manner if NAT10 can directly affect microtubule dynamics, we performed NAT10 IP followed by mass spectrometry to investigate whether NAT10 directly interacts with cytoskeletal proteins in control and/or FTD-MAPT neurons (Figure 6C). In neurons of each genotype, in addition to NAT10 itself, known NAT10-interacting proteins involved in RNA processing were identified, including THUMPD1 and several ribosomal proteins (Figure 6C). In addition, several microtubule-associated and microtubule-regulating proteins were identified, including MAP1, AGAP1, AGAP3, SPECC1, KIF2A, and ARHGEF2. Interaction of NAT10 with KIF2A and ARHGEF2 was further confirmed by NAT10 immunoprecipitation followed by immunoblotting (Figure 6D). Collectively, these data demonstrate that NAT10 interacts with several proteins involved in regulating microtubules and suggest that one mechanism by which NAT10 inhibition corrects nuclear membrane abnormalities is by modulating cytoskeletal dynamics.

## Discussion

We report here that the inhibition or loss of function of the acetyltransferase NAT10 suppresses the pathogenic effect of tau in human FTD-MAPT neurons *in vitro* and in a *Drosophila* tauopathy model *in vivo*. In both human FTD-MAPT and *Drosophila MAPT* R406W neurons, nuclear lamina defects were reversed by small-molecule inhibition of NAT10 and by reduction of NAT10 protein levels. NAT10 inhibition or haploinsufficiency in a *Drosophila* tauopathy model reduced the neurotoxic effects of human mutant tau expression and extended lifespan. Correction of nuclear lamina defects in human FTD neurons reversed defective nucleocytoplasmic transport. Inhibition of NAT10 induced changes in microtubule dynamics specifically in FTD-MAPT, but not healthy control, neurons. In addition, unbiased proteomic analyses identified several regulators of microtubule dynamics as NAT10-interacting proteins, two of which, KIF2A and ARHGEF2, suggested that the inhibition of NAT10 corrects nuclear membrane function by correcting abnormal microtubule dynamics.

Previously, we reported that tau mutations causal for FTD led to tau accumulation in the neuronal cell body, nuclear lamina defects, and perturbed nucleocytoplasmic transport.^4^ Nocodazole-induced microtubule depolymerization acutely corrected nuclear lamina deformation, as we also observed in HGPS fibroblasts.^13^ We also found that treatment of HGPS fibroblasts with a small-molecule inhibitor of NAT10, Remodelin, corrects nuclear lamina defects, at least in part by releasing microtubule forces exerted on the nucleus.^13^ Given the similarities in nuclear phenotypes between HGPS and FTD-MAPT, we investigated whether inhibition or loss of function of NAT10 could reverse phenotypes in FTD-MAPT neurons.

We report here that NAT10 inhibition corrected nuclear lamina defects in human FTD-MAPT neurons. We extended these findings to an *in vivo* model of tauopathy, demonstrating that NAT10 inhibition and haploinsufficiency both significantly ameliorate nuclear shape abnormalities in a *Drosophila* model of tauopathy, *MAPT* R406W, where nuclear envelope defects have been previously reported.^9^ Highlighting the functional relevance of correcting tau-mediated pathologies, NAT10 inhibition and haploinsufficiency extended the lifespan of tauopathy model animals significantly.

Consistent with NAT10 inhibition having acute effects on neuronal cell biology, microtubule elongation was reduced in neurons within 48 h of drug treatment, with no effects on levels of tau protein or several key cytoskeletal components, including tubulin and actin. Furthermore, microtubule dynamics were only altered by NAT10 inhibition in FTD-MAPT neurons, with no effect on non-demented and isogenic control neurons. The widely accepted canonical function of tau protein is to stabilize axonal microtubules; however, tau can also promote microtubule polymerization.^19^ It is possible that accumulation of tau in the neuronal cell body results in a gain-of-function effect on microtubule dynamics specifically in FTD-MAPT neurons, promoting microtubule formation in the neuronal cell body, altering its elongation and enabling transfer of mechanical forces to the nuclear membrane, deforming the nuclear lamina. Notably, interactions between tau protein aggregates and the nuclear pore complex have been detected both *in vitro* and *in vivo.*^7^ Therefore, another mechanism for tau’s disruption of the nuclear membrane is via tau-mediated coupling of microtubules to the nuclear pore complex. In each scenario, abnormal neuronal cell-body microtubule dynamics, promoted by tau accumulation, could disrupt the nuclear membrane directly via LINC complex-mediated microtubule-nuclear envelope interactions.^20^ Given that these changes are specific to FTD-MAPT neurons, NAT10 inhibition would be predicted to only alter microtubule dynamics in FTD-MAPT, and not healthy control, neurons.

The acetyltransferase NAT10 is well characterized as a cytidine acetyltransferase that modifies several different forms of RNA, including ribosomal and tRNAs and protein-coding mRNAs.^21^ However, NAT10 also acetylates a number of proteins, including histones, p53, and alpha-tubulin.^22^^,^^23^^,^^24^ There is increasing evidence for a role for NAT10 in regulating microtubule dynamics. We have previously found that NAT10 acetylates tubulin and that this can be inhibited by Remodelin.^13^ Furthermore, NAT10 regulates microtubule spindle assembly in mitotic cells via acetylation of Eg5/KIF11, a kinesin that cross-links adjacent microtubules, enabling anti-parallel sliding.^25^ KIF11 is expressed in neurons, where it regulates microtubule dynamics and dendritic growth.^26^ Thus, NAT10 can directly enhance microtubule stability via tubulin acetylation, a modification that promotes microtubule flexibility and overall stability,^27^ and indirectly enable microtubule dynamics via acetylation of KIF11.

We extend those findings here, where we find that NAT10 interacts with several regulators of the microtubule cytoskeleton, including the kinesin KIF2A and the guanosine triphosphate (GTP) exchange factor ARHGEF2. KIF2A primarily depolymerizes microtubules but under specific conditions can also stabilize microtubules.^28^ ARHGEF2 directly binds to microtubules and has been characterized as signaling the assembly state of microtubules, leading to changes in the actin-myosin network.^29^ KIF2A and ARHGEF2 have both been identified as tau-interacting proteins, with ARHGEF2 showing higher levels of tau association in postmortem samples from rapidly progressive AD.^30^^,^^31^ These interactions support a role for NAT10 in regulating cytoskeletal dynamics in human neurons, including indirectly interacting with the tau protein. Loss of NAT10 function would be predicted to reduce microtubule flexibility, stability, and motility, relieving deforming forces on the nuclear envelope, as we have previously observed with acute microtubule depolymerization with nocodazole.^4^

Overall, this study highlights the importance of nuclear lamina integrity and microtubule dynamics in the pathology of FTD and other tauopathies and proposes a novel therapeutic approach to target these cellular processes: rescue of abnormal microtubule dynamics. Further research is required to fully understand the mechanisms by which inhibition of NAT10 exerts its effects and to explore its potential as a treatment for neurodegenerative diseases characterized by tau pathology. Future studies will explore the long-term effects of NAT10 inhibition or KD *in vivo* and investigate whether these cellular improvements translate into functional benefits and neuroprotection in other animal models of tauopathies.

### Limitations of the study

Our study has several limitations. First, while we report correction of nuclear morphology and nucleocytoplasmic transport following NAT10 inhibition, we did not directly assess whether these improvements lead to enhanced neuronal function or protection against neurodegeneration in human neurons. Furthermore, we observed that complete NAT10 loss appears lethal in both human iPSCs and *Drosophila*, suggesting a narrow therapeutic window for NAT10 inhibition. Future studies should therefore investigate the long-term consequences of sustained NAT10 inhibition/downregulation on human neuronal viability and function. In addition, our *in vivo* studies were limited to *Drosophila* models. Although these experiments demonstrated beneficial effects of NAT10 inhibition on nuclear morphology and lifespan, validation in mammalian tauopathy models will be necessary to establish translational relevance, and to evaluate the long-term efficacy and safety of NAT10 inhibition in the nervous system. Finally, the mechanistic link between NAT10 activity and microtubule dynamics remains incompletely resolved. Although Remodelin altered EB3 dynamics in FTD-MAPT neurons and NAT10 interacted with several regulators of microtubule dynamics, the molecular mechanisms underlying these effects were not directly investigated. Additional studies should therefore explore the functional role of NAT10 interactions with proteins such as KIF2A and ARHGEF2 in regulating the neuronal cytoskeleton.

## Resource availability

### Lead contact

Requests for further information and resources should be directed to the lead contact, Frederick J. Livesey (rick@talisman-therapeutics.com).

### Materials availability

Plasmid generated in this study is available from the lead contact with a completed materials transfer agreement.

### Data and code availability

• Data reported in this paper will be shared by the lead contact upon request.
• The study in this article does not report original code.
• Any additional information required to reanalyze the data reported in this paper is available from the lead contact upon request.

## Acknowledgments

The authors wish to thank our colleagues at the Gurdon Institute, UCL, and Talisman for ongoing support. This work was funded by a Wellcome Trust Senior Investigator Award (WT101052MA), 10.13039/501100002283Alzheimer’s Research UK (funding for Stem Cell Research Centre), and Great Ormond Street Children’s Charity Professorship to F.J.L. and a Wellcome Trust Senior Investigator Award (103792), Wellcome Trust Investigator Award (223111), and Royal Society Darwin Trust Research Professorship (RSRP\R\210002) to A.H.B. A.H.B. acknowledges core funding to the Gurdon Institute from the Wellcome Trust (092096) and 10.13039/501100000289CRUK (C6946/A14492). D.L. was funded by a Sir Henry Dale Fellowship jointly funded by the Wellcome Trust and the 10.13039/501100000288Royal Society 206242/Z/17/Z.

## Author contributions

The study was designed and planned by F.P., A.H.B., and F.J.L. The experimental work was performed by F.P., B.D.B., T.C., E.C., J.L., R.S., J.S., and C.M.D. The image analysis pipeline was assembled by R.B. All authors contributed to data analysis, figure preparation, and manuscript drafting.

## Declaration of interests

D.L. is an employee of Altos Labs. F.J.L. is a founder, shareholder, and employee of Talisman Therapeutics. F.P., J.S., E.C., and T.C. are employees of Talisman Therapeutics. R.B. is a consultant for Talisman Therapeutics. B.D.B. is a founder, quota holder, and employee of NBTX GmbH.

## STAR★Methods

### Key resources table

**Table:** 

REAGENT or RESOURCE	SOURCE	IDENTIFIER
**Antibodies**		

Anti-NAT10 Polyclonal antibody	Proteintech	Cat#13365-1-AP; RRID:AB_2148944
Anti-Kif2a antibody	Abcam	Cat#ab197988; RRID:AB_2921234
Anti-GEF H1 antibody	Thermo Fisher Scientific	Cat#PA5-32213; RRID:AB_2549686
Anti-LaminB1; rabbit polyclonal	Abcam	Cat#ab16048; RRID:AB_443298
Anti-total tau; rabbit polyclonal	Dako Cytomation	Cat#A0024; RRID:AB_10013724
Anti-β-actin; mouse monoclonal	Sigma	Cat#A2228; RRID:AB_476697
Anti-β3-tubulin; mouse monoclonal	BioLegend	Cat#MMS-435P; RRID:AB_2313773
Anti-Phospho-tau pS202/T205 - AT8; mouse monoclonal	Thermo Fisher Scientific	Cat#MN1020; RRID:AB_223647
Anti-Phospho-Tau (Thr181) mouse Monoclonal Antibody (AT270)	Thermo Fisher Scientific	Cat#MN1050; RRID:AB_223651
Anti-Human Tau, phospho (Thr217) rabbit Polyclonal Antibody, Unconjugated	Thermo Fisher Scientific	Cat#44–744; RRID:AB_2533741
Anti-Acetyl-alpha Tubulin (Lys40) mouse monoclonal Antibody (6-11B-1)	Thermo Fisher Scientific	Cat#32–2700; RRID:AB_2533073
Anti-α-tubulin	Abcam	Cat# ab15246; RRID:AB_301787
Anti-Cofilin 1 mouse monoclonal	Bio-Rad	Cat#VMA00870
Anti-Cofilin (D3F9) XP rabbit monoclonal	Cell Signaling Technology	Cat#2175; RRID:AB_10622000
Anti-Lamin A/C mouse monoclonal (mab636)	Thermo Fisher Scientific	Cat#MA3-1000; RRID:AB_325377
Alexa Fluor 488 donkey anti-rabbit	Thermo Fisher Scientific	Cat#A21206; RRID:AB_2535792
Alexa Fluor 647 donkey anti-mouse	Thermo Fisher Scientific	Cat#A-31571; RRID:AB_162542
Alexa Fluor 568 donkey anti-chicken	Thermo Fisher Scientific	Cat# A78950; RRID:AB_2921072
Alexa Fluor 488 Goat anti-Mouse	Thermo Fisher Scientific	Cat# A-10680, RRID:AB_2534062
Anti-MAP2 chicken polyclonal	Abcam	Cat#ab5392; RRID:AB_2138153
Normal Rabbit IgG	Cell Signaling Technology	Cat#2729; RRID:AB_1031062
Anti-Drosophila Lamin Dm0 mouse monoclonal	Developmental Studies Hybridoma Bank	Cat# ADL67.10; RRID:AB_528336

**Chemicals, peptides, and recombinant proteins**		

2-Mercaptoethanol	Thermo Fisher Scientific	Cat#21985-023
Accutase	Innovative Cell Technologies	Cat#AT104
B-27 supplement	Thermo Fisher Scientific	Cat#17504-044
Bovine Serum Albumin (BSA)	Sigma	Cat#A2153
cOmplete, Mini, EDTA-free Protease Inhibitor Cocktail Tablets	Sigma	Cat#04693159001
DAPI	Sigma	Cat#D9542
Dispase	Thermo Fisher Scientific	Cat#17105
DMEM/F-12, GlutaMAX	Thermo Fisher Scientific	Cat#31331-028
DNase I	New England BioLabs	Cat#M0303S
Dimethyl sulfoxide (DMSO)	Sigma	Cat#D2650
Dorsomorphin dihydrochloride	Tocris	Cat#3093
Essential 8 medium	Thermo Fisher Scientific	Cat#A1517001
Fibroblast growth factor 2 (FGF2)	PeproTech	Cat#100-18B
Halt Phosphatase Inhibitor Cocktail	Thermo Fisher Scientific	Cat#78420
High Capacity cDNA Reverse Transcription kit	Thermo Fisher Scientific	Cat#4368814
PowerUp™ SYBR™ Green Master Mix for qPCR	Thermo Fisher Scientific	Cat# A25741
Insulin	Sigma	Cat#19278
L-Glutamine	Thermo Fisher Scientific	Cat#25030-024
Laminin	Sigma	Cat#L2020
*N*-2 supplement	Thermo Fisher Scientific	Cat#17502-048
Neurobasal	Thermo Fisher Scientific	Cat#12348-017
Non-essential amino acid solution	Thermo Fisher Scientific	Cat#11140-050
NuPAGE LDS Sample Buffer	Thermo Fisher Scientific	Cat#NP0007
Paraformaldehyde (PFA)	Sigma	Cat#158127
Dulbecco’s Phosphate Buffered Saline	Merck	Cat#D8537
DPBS, calcium, magnesium	Thermo Fisher Scientific	Cat#14040133
Penicillin-streptomycin	Thermo Fisher Scientific	Cat#15140-122
RIPA Buffer	Sigma	Cat#R0278
SB431542	Tocris Bioscience	Cat#1614
Sodium pyruvate	Sigma	Cat#S8636
Triton X-100	Sigma	Cat#T8787
RNeasy Mini Kit	Qiagen	Cat# 74104
Tween 20	Sigma	Cat#P9416
Tween 80	Sigma	Cat#P8074
2-hydroxypropyl-b-cyclodextrin solution	Sigma	Cat#H5784
Remodelin hydrobromide	Merck	Cat#SML1112
Accell siRNA Delivery Media	Horizon Discovery	Cat#B-005000-100
Recombinant NAT10 protein	Active Motif	Cat#81376
Accell Human NAT10 siRNA, SMARTPool 20 nmol	Horizon Discovery	Cat# E−014402-00-0020
Accell Non-targeting Control Pool 20 nmol	Horizon Discovery	Cat#D-001910-10-20
Phalloidin-iFluor 647 Reagent	Abcam	Cat#ab176759
RLT lysis buffer	Qiagen	Cat#79216

**Critical commercial assays**		

BCA Protein Assay Kits	Thermo Fisher Scientific	23225
4–15% Mini-PROTEAN® TGX Stain-Free™ Protein Gels	Bio-Rad	4568083
Trans-Blot Turbo Transfer Pack	Bio-Rad	1704157

**Experimental models: Cell lines**		

Human: non-demented control (NDC) iPSC line	Israel et al.^32^	N/A
Human: MAPT IVS10 + 16-A iPSC line	StemBANCC	SFC850.03.01
Human: MAPT IVS10 + 16-B iPSC line	StemBANCC	SFC851.03.04

**Experimental models: Organisms/strains**		

Drosophila:	N/A	N/A
y[1] w[67c23] P{w[+mC] = lacW}l(1)G0020[G0020]/FM7c	BDSC	RRID:BDSC_11474
w[∗]; +; UAS MAPT 0N4R R406W	Frost et al.^6^	N/A
w[∗]; P{w[+mC] = GAL4-elav.L}CG16779[3]	BDSC	RRID:BDSC_8760

**Oligonucleotides**		

NAT10 F 5′-GGATTGCCTCAACATCACTCGG-3′	This paper	N/A
NAT10 R 5′-CGTTGGAGGAAAACTTCAGAGGC-3′	This paper	N/A
GAPDH F 5′-GTCTCCTCTGACTTCAACAGCG-3′	This paper	N/A
GAPDH R 5′-ACCACCCTGTTGCTGTAGCCAA-3′	This paper	N/A

**Recombinant DNA**		

pSyn1:EB3-GFP	This paper	N/A
S-tdTomato	Jeffrey Rothstein	Addgene plasmid # 112579; RRID:Addgene_112579

**Software and algorithms**		

ABI StepOnePlus software	Thermo Fisher Scientific	N/A; RRID:N/A
Fiji	Schindelin et al.^33^	RRID:SCR_002285
plusTipTracker	Applegate et al.^34^	N/A; RRID:N/A
Leica FRAP-wizard tool	Leica	N/A; RRID: N/A
Harmony High-Content Imaging and Analysis Software	Revvity	Cat#HH17000019
Prism 10	GraphPad	RRID:SCR_002798
Mascot	MATRIX Science	RRID:SCR_014322

**Other**		

μ-Dish 35 mm, high	ibidi	Cat#81156

### Experimental model and study participant details

Generation of the *MAPT* IVS10 + 16-A and *MAPT* IVS10 + 16-B mutant iPSCs was previously reported^35^ and these lines were obtained from the EU StemBANCC consortium (reference numbers SFC850.03.01 and SFC851.03.04 respectively) with ethics approval 09/H0716/64. Genotype was confirmed upon receipt by Sanger sequencing of the *MAPT* intron 10. The non-demented control line was previously reported.^32^ The isogenic revertant of the *MAPT* IVS10 + 16-B iPSC line was generated by CRISPR/Cas9 editing of the *MAPT* IVS10 + 16-B line, reverting the mutant allele to the wild-type allele. The *MAPT* IVS10 + 16-A, *MAPT* IVS10 + 16-B and isogenic *MAPT* IVS10 + 16-B line were XX karyotype, the non-demented control line was XY karyotype. Previous studies have reported that there are not sex differences in the prevalence of FTD due to mutations in *MAPT*,^36^ therefore iPSCs of different sex were not compared in this study. iPSC lines used in this study were tested and found negative for mycoplasma contamination. *Drosophila* carrying UAS-*MAPT* 0N4R R406W (kindly donated by Mel Feany) were crossed to w1118; +; elaV-GAL4 (Bloomington Stock Center #8760) for pan-neuronal expression of *MAPT* R406W.^6^^,^^9^ w1118 animals were used as controls. l(1)G00020 mutant animals (Bloomington Stock Center #11474; dNAT10 with P{lacW} insertion at the transcriptional start site) were crossed to generate the l(1)G0020/FM7c; +; elaV-GAL4 line. All studies were carried out on mixed populations of adult male and female flies, all of which were maintained on standard cornmeal-agar medium.

### Method details

#### Generation of iPSC-derived cortical neurons and drug/siRNA treatments

iPSC cells were grown and expanded in feeder-free conditions using Essential 8 Medium (Thermo Fisher Scientific), at 37°C with 5% CO_2_. Essential 8 Medium was replaced daily, and cells were routinely passaged using 0.5 mM EDTA when 60–80% confluent. Differentiation of iPSCs to cortical neurons was carried out as described, with minor modifications.^37^^,^^38^ Differentiated neurons were maintained in culture for up to 120 days. To establish identity and quality of cortical neuronal inductions, gene expression profiling was performed on a custom gene expression panel of approximately 250 genes.^39^ After subtracting the maximum negative control probe counts, gene counts were normalized using the geometric mean of 6 positive control probes and 7 housekeeping genes (CLTC, GAPDH, GUSB, PPIA, RPLP1, RPS15A, RPS9).

For Remodelin hydrobromide (Merck) treatment, neurons were grown for 120 days *in vitro* (DIV) and compound was added at 7.5 μM before imaging. DMSO was used as vehicle. For siRNA treatment, neurons were grown to 80 DIV and siRNA targeting NAT10 (Accell Human NAT10 siRNA, SMARTPool; Horizon Discovery) or scramble siRNA (Accell Non-targeting Control Pool; Horizon Discovery) were added at 1 μM in minimal media (Accell siRNA Delivery media; Horizon Discovery) for 24h before being supplemented with neuronal maintenance media for a further 48h. This 3-day siRNA protocol was repeated twice to achieve maximal siRNA KD.

#### Protein extraction, western blot analysis and co-immunoprecipitation

Total cell protein was extracted with RIPA buffer (Sigma) supplemented with protease inhibitors (Sigma) and Halt phosphatase inhibitors (Thermo Fisher Scientific). Samples were run on 4–15% Mini-PROTEAN TGX Stain-Free Protein Gels (Bio-Rad) and proteins transferred to PVDF membrane using a Trans-Blot Turbo Transfer Pack (Bio-Rad). All primary antibodies were incubated overnight in 5% BSA in PBS+ 0.1% Tween 20 (PBST) at 4°C. For detection, membranes were incubated for at least 1h in secondary antibody, washed in PBST buffer and imaged on a Bio-Rad ChemiDoc Imaging System. For co-immunoprecipitation experiments, 1 mg of total protein was immunoprecipitated using a polyclonal anti-NAT10 antibody (Proteintech) or normal Rabbit IgG (Cell Signaling). Immunoprecipitated samples were then analyzed by western blot as described above using a polyclonal anti-KIF2A antibody (abcam) or a polyclonal Anti-ARHGEF2 (abcam).

#### Antibodies used in the study

The following antibodies were used in the study: anti-LaminB1 (abcam, ab16048), anti-total tau (Dako Cytomation, A0024), anti- Phospho-tau pS202/T205 - AT8 (Thermo Fisher Scientific, MN1020), anti- Phospho-Tau pThr181 (Thermo Fisher Scientific, MN1050), anti- Phospho-Tau pThr217 (Thermo Fisher Scientific, 44–744), anti- β3-tubulin (BioLegends, MMS-435P), anti-NAT10 (Proteintech, 13365-1-AP), anti-KIF2A (abcam, A37944), amti-ARHGEF2 (Thermo Fisher Scientific, PA5-32213), anti-acTubulin K40 (Thermo Fisher Scientific, 322700), anti-α-tubulin (abcam, ab15246), anti-Cofilin 1 mouse monoclonal (Bio-Rad, VMA00870), anti-Cofilin (D3F9) XP rabbit monoclonal (Cell Signaling, 2175), Anti-Lamin A/C (Thermo Fisher Scientific, MA3-1000).

#### Immunofluorescence and imaging – human neurons

2D cultured cells on optically appropriate tissue culture plates were washed twice in PBS prior to incubation with 4% PFA in PBS for 15 min at room temperature. Cells were washed twice with PBS and permeabilized in PBS+0.3% Triton X-100. Permeabilized cells were then incubated in blocking medium (5% v/v solution of BSA in PBS with 0.3% Triton X-) before primary antibody addition and incubation overnight at 4°C. Following primary antibody labeling, secondary antibodies were added in the presence of DAPI (Sigma Aldrich, D9542). Immunostained samples were kept in PBS and stored at 4°C prior to imaging.

#### Nuclear shape scoring – iPSC-derived neurons

Nuclear shape was scored as previously described.^4^ Briefly, samples were fixed and stained for Lamin B1 and DAPI before imaging. Images were scored using an unbiased custom Fiji script where DAPI signal is used to identify the area occupied by each nucleus and LaminB1 signal was assigned as either proximal to DAPI boundary or invaginated (i.e., within the nuclear area). The proportion of invaginated laminB1 was calculated relative to the total laminB1 signal per nucleus. Based on the distribution of invagination scores in control (wild-type) nuclei, nuclei with invagination scores >0.3 were empirically defined as invagination+. For nuclear solidity scoring, DAPI signal was used to define nuclear shape, and a custom unbiased Fiji script was used to score area convex hull. Solidity is reported as Nuclear Area/Nuclear Convex hull (Figure S1C).

#### qRT-PCR

Cultures were lysed using RLT lysis buffer and RNA purification was performed using an RNeasy Mini Kit (QIAGEN), according to manufacturer’s protocol. 500 ng of purified RNA was reverse transcribed into cDNA using a High-Capacity cDNA Reverse Transcription Kit (Thermo), according to manufacturer’s protocol. 10 ng cDNA was analysed by qRT-PCR using PowerUp SYBR Green Master Mix (Thermo) on a StepOnePlus Real-Time PCR System (Thermo), according to manufacturer’s protocol, using the following primer pairs: NAT10 F 5′-GGATTGCCTCAACATCACTCGG-3'; NAT10 R 5′-CGTTGGAGGAAAACTTCAGAGGC-3'; GAPDH F 5′-GTCTCCTCTGACTTCAACAGCG-3'; GAPDH R 5′-ACCACCCTGTTGCTGTAGCCAA-3'. All NAT10 expression data were normalised to GAPDH expression and primer-specificity was confirmed at the end of each qRT-PCR run via the generation of single peaks in melt-curve analyses. Data analysis was performed using the 2-ΔΔCT method.

#### *Drosophila* food preparation

Fresh, liquid standard cornmeal-agar medium was kept in a water bath at 55°C. 4 mL of liquid medium was transferred into vials and either 16 μL vehicle (20% DMSO; 65% 2-hydroxypropyl-β-cyclodextrin solution, H5784 Sigma Aldrich; 15% Tween 80, P8074 Sigma Aldrich^11^; or 16 μL Remodelin 25 mM stock solution (100 μM final Remodelin food concentration) was added to the liquid cornmeal-agar food, thoroughly mixed and stored overnight in a dark chamber at 4°C before bringing to room temperature. Remodelin food ingestion was confirmed by red dye food staining (Figure S2).

#### Nuclear shape scoring - *Drosophila*

Nuclear shape was scored based on Lamin B (Developmental Studies Hybridoma Bank, ADL67.10) immunostaining. Nuclear morphology was scored according to features described.^40^ Confocal imaging of 4% PFA-fixed brain samples was performed using a Leica SP8 confocal microscope with a 63× oil-immersion objective. Brains were imaged on the ventral side of the antennal lobe at 7 μM depth, whereas max. one section was sampled and scored per brain hemisphere. Images were anonymized via a macro and subsequently scored with the open-source software Fiji.^33^

#### Lifespan

Lifespan assays were performed according to the protocol described.^41^ 5 replicates of equal-sized groups of animals per condition and genotype (25–35 females) were transferred to food vials and maintained at 25°C in a 12:12 light-dark humidity-controlled incubator. Animals were transferred to fresh food vials every 3–4 days and dead animals were scored every 1–2 days.

#### Live imaging of microtubule dynamics

Neurons were grown to 100 DIV in individual m-Dish 35 mm dishes (Ibidi) and transduced with a lentivirus encoding for GFP-EB3 driven by Synapsin 1 promoter (derived from plasmid provided by Michael Davidson; Addgene plasmid # 56474). 48h after transfection, neurons were subjected to live imaging using a Leica SP5 microscope equipped with a controlled environment chamber (37°C; 5% CO_2_). Images were acquired at resonant scanning with a 63× objective (1frame/sec). Resulting movies were analyzed using the plusTipTracker software.^34^

#### Nucleocytoplasmic transport assay

Nucleocytoplasmic trafficking was analyzed by infection of 120 DIV human iPSC-derived neurons with the lentiviral CMV–NLS–tdTomato–NES construct. Two days post infection, iPSC neurons were subjected to FRAP analysis using a Leica SP8 inverted confocal microscope equipped with a live imaging chamber (Okolab). Signal from tdTomato was recorded every 0.3 s for 15 s before being bleached in the nuclear area, for 30 iterations of 40–60% laser power. Recovery was monitored every 0.3 s for 150 s. Recovery was normalized to the average of the pre-bleached signals. Results were elaborated using the Leica FRAP-wizard tool. Lentiviral-S-tdTomato was a gift from Jeffrey Rothstein (Addgene plasmid # 112579; http://n2t.net/addgene:112579; RRID:Addgene_112579).

#### Immunoprecipitation and mass spectrometric analysis of NAT10 interacting proteins

NAT10 was immunoprecipitated from 1.4 mg of total protein extracted from iPSC-derived neurons (120 DIV) using a polyclonal anti-NAT10 antibody (Proteintech). Immunoprecipitated samples were used for subsequent mass spectrometry analysis. Peptide masses of digested protein samples were determined using a Bruker ultrafleXtreme Maldi mass spectrometer in reflectron mode and ms/ms fragmentation performed in LIFT mode. Data analysis was with FlexAnalysis, BioTools and ProteinScape software (Bruker). Database searches of the combined mass fingerprint-ms/ms data were performed using Mascot (http://www.matrixscience.com).

### Quantification and statistical analysis

Data are presented as mean or median values of the number of independently conducted experiments indicated in the legend of each figure. Unless otherwise indicated, error bars represent the standard deviation (SD). Statistical analysis was performed using the GraphPad Prism 11.0.0 analytical software (GraphPad Software). Unpaired Student’s *t* test was used to compare differences between two groups, assuming the data were normally distributed. One-way ANOVA or two-way ANOVA followed by specified correction for multiple testing was used to analyze the differences between more than two groups. For survival experiments, the Kaplan-Meier method was used to generate survival curves and statistical significance was assessed with the log rank test. Hazard ratios with 95% confidence intervals were calculated using Cox proportional hazards regression. The ‘‘n’’ for each individual experiment is listed in the figure legends. Asterisks (∗, ∗∗ and ∗∗∗) indicate *p* < 0.05, 0.01 and 0.001, respectively.

## Supplemental information

Document S1. Figures S1–S5

Document S2. Paonessa uncropped blots

Table S1. Mass spectrometry data from NAT10 IP-MS study

## References

[1] Creekmore B.C., Watanabe R., Lee E.B. (2024). Neurodegenerative Disease Tauopathies. Annu. Rev. Pathol..

[2] Goedert M., Crowther R.A., Scheres S.H.W., Spillantini M.G. (2024). Tau and neurodegeneration. Cytoskeleton (Hoboken).

[3] Ghetti B., Oblak A.L., Boeve B.F., Johnson K.A., Dickerson B.C., Goedert M. (2015). Invited review: Frontotemporal dementia caused by microtubule-associated protein tau gene (MAPT) mutations: a chameleon for neuropathology and neuroimaging. Neuropathol. Appl. Neurobiol..

[4] Paonessa F., Evans L.D., Solanki R., Larrieu D., Wray S., Hardy J., Jackson S.P., Livesey F.J. (2019). Microtubules Deform the Nuclear Membrane and Disrupt Nucleocytoplasmic Transport in Tau-Mediated Frontotemporal Dementia. Cell Rep..

[5] Fernández-Nogales M., Cabrera J.R., Santos-Galindo M., Hoozemans J.J.M., Ferrer I., Rozemuller A.J.M., Hernández F., Avila J., Lucas J.J. (2014). Huntington’s disease is a four-repeat tauopathy with tau nuclear rods. Nat. Med..

[6] Frost B., Hemberg M., Lewis J., Feany M.B. (2014). Tau promotes neurodegeneration through global chromatin relaxation. Nat. Neurosci..

[7] Eftekharzadeh B., Daigle J.G., Kapinos L.E., Coyne A., Schiantarelli J., Carlomagno Y., Cook C., Miller S.J., Dujardin S., Amaral A.S. (2018). Tau Protein Disrupts Nucleocytoplasmic Transport in Alzheimer’s Disease. Neuron.

[8] Cornelison G.L., Levy S.A., Jenson T., Frost B. (2019). Tau-induced nuclear envelope invagination causes a toxic accumulation of mRNA in Drosophila. Aging Cell.

[9] Frost B., Bardai F.H., Feany M.B. (2016). Lamin Dysfunction Mediates Neurodegeneration in Tauopathies. Curr. Biol..

[10] Pollex R.L., Hegele R.A. (2004). Hutchinson-Gilford progeria syndrome. Clin. Genet..

[11] Balmus G., Larrieu D., Barros A.C., Collins C., Abrudan M., Demir M., Geisler N.J., Lelliott C.J., White J.K., Karp N.A. (2018). Targeting of NAT10 enhances healthspan in a mouse model of human accelerated aging syndrome. Nat. Commun..

[12] Larrieu D., Viré E., Robson S., Breusegem S.Y., Kouzarides T., Jackson S.P. (2018). Inhibition of the acetyltransferase NAT10 normalizes progeric and aging cells by rebalancing the Transportin-1 nuclear import pathway. Sci. Signal..

[13] Larrieu D., Britton S., Demir M., Rodriguez R., Jackson S.P. (2014). Chemical inhibition of NAT10 corrects defects of laminopathic cells. Science.

[14] McGurk L., Berson A., Bonini N.M. (2015). Drosophila as an In Vivo Model for Human Neurodegenerative Disease. Genetics.

[15] Wittmann C.W., Wszolek M.F., Shulman J.M., Salvaterra P.M., Lewis J., Hutton M., Feany M.B. (2001). Tauopathy in Drosophila: neurodegeneration without neurofibrillary tangles. Science.

[16] Gistelinck M., Lambert J.-C., Callaerts P., Dermaut B., Dourlen P. (2012). Drosophila Models of Tauopathies: What Have We Learned?. Int. J. Alzheimers Dis..

[17] Kim H.J., Taylor J.P. (2017). Lost in transportation: nucleocytoplasmic transport defects in ALS and other neurodegenerative diseases. Neuron.

[18] Zhang K., Donnelly C.J., Haeusler A.R., Grima J.C., Machamer J.B., Steinwald P., Daley E.L., Miller S.J., Cunningham K.M., Vidensky S. (2015). The C9orf72 repeat expansion disrupts nucleocytoplasmic transport. Nature.

[19] Drubin D.G., Kirschner M.W. (1986). Tau protein function in living cells. J. Cell Biol..

[20] Kalukula Y., Stephens A.D., Lammerding J., Gabriele S. (2022). Mechanics and functional consequences of nuclear deformations. Nat. Rev. Mol. Cell Biol..

[21] Achour C., Oberdoerffer S. (2024). NAT10 and cytidine acetylation in mRNA: intersecting paths in development and disease. Curr. Opin. Genet. Dev..

[22] Dalhat M.H., Altayb H.N., Khan M.I., Choudhry H. (2021). Structural insights of human N-acetyltransferase 10 and identification of its potential novel inhibitors. Sci. Rep..

[23] Liu X., Tan Y., Zhang C., Zhang Y., Zhang L., Ren P., Deng H., Luo J., Ke Y., Du X. (2016). NAT10 regulates p53 activation through acetylating p53 at K120 and ubiquitinating Mdm2. EMBO Rep..

[24] Shen Q., Zheng X., McNutt M.A., Guang L., Sun Y., Wang J., Gong Y., Hou L., Zhang B. (2009). NAT10, a nucleolar protein, localizes to the midbody and regulates cytokinesis and acetylation of microtubules. Exp. Cell Res..

[25] Zheng J., Tan Y., Liu X., Zhang C., Su K., Jiang Y., Luo J., Li L., Du X. (2022). NAT10 regulates mitotic cell fate by acetylating Eg5 to control bipolar spindle assembly and chromosome segregation. Cell Death Differ..

[26] Freixo F., Martinez Delgado P., Manso Y., Sánchez-Huertas C., Lacasa C., Soriano E., Roig J., Lüders J. (2018). NEK7 regulates dendrite morphogenesis in neurons via Eg5-dependent microtubule stabilization. Nat. Commun..

[27] McKenna E.D., Sarbanes S.L., Cummings S.W., Roll-Mecak A. (2023). The Tubulin Code, from Molecules to Health and Disease. Annu. Rev. Cell Dev. Biol..

[28] Stockmann L., Kabbech H., Kremers G.-J., van Herk B., Dille B., van den Hout M., van IJcken W.F.J., Dekkers D.H.W., Demmers J.A.A., Smal I. (2025). KIF2A stabilizes intercellular bridge microtubules to maintain mouse embryonic stem cell cytokinesis. J. Cell Biol..

[29] Mitchison T.J. (2026). Dynamic microtubules as sensors in animal cells. Biophys. J..

[30] Atwa A., Alhadidy M.M., Lamp J., Combs B., Kanaan N.M. (2025). BioID2-Based Tau Interactome Reveals Novel and Known Protein Interactions Associated with Multiple Cellular Pathways. J. Proteome Res..

[31] Younas A., Younas N., Iqbal M.J., Ferrer I., Zerr I. (2024). Comparative interactome mapping of Tau-protein in classical and rapidly progressive Alzheimer’s disease identifies subtype-specific pathways. Neuropathol. Appl. Neurobiol..

[32] Israel M.A., Yuan S.H., Bardy C., Reyna S.M., Mu Y., Herrera C., Hefferan M.P., Van Gorp S., Nazor K.L., Boscolo F.S. (2012). Probing sporadic and familial Alzheimer’s disease using induced pluripotent stem cells. Nature.

[33] Schindelin J., Arganda-Carreras I., Frise E., Kaynig V., Longair M., Pietzsch T., Preibisch S., Rueden C., Saalfeld S., Schmid B. (2012). Fiji: an open-source platform for biological-image analysis. Nat. Methods.

[34] Applegate K.T., Besson S., Matov A., Bagonis M.H., Jaqaman K., Danuser G. (2011). plusTipTracker: Quantitative image analysis software for the measurement of microtubule dynamics. J. Struct. Biol..

[35] Sposito T., Preza E., Mahoney C.J., Setó-Salvia N., Ryan N.S., Morris H.R., Arber C., Devine M.J., Houlden H., Warner T.T. (2015). Developmental regulation of tau splicing is disrupted in stem cell-derived neurons from frontotemporal dementia patients with the 10 + 16 splice-site mutation in MAPT. Hum. Mol. Genet..

[36] Curtis A.F., Masellis M., Hsiung G.-Y.R., Moineddin R., Zhang K., Au B., Millett G., Mackenzie I., Rogaeva E., Tierney M.C. (2017). Sex differences in the prevalence of genetic mutations in FTD and ALS. Neurology.

[37] Shi Y., Kirwan P., Livesey F.J. (2012). Directed differentiation of human pluripotent stem cells to cerebral cortex neurons and neural networks. Nat. Protoc..

[38] Shi Y., Kirwan P., Smith J., Robinson H.P.C., Livesey F.J. (2012). Human cerebral cortex development from pluripotent stem cells to functional excitatory synapses. Nat. Neurosci..

[39] Strano A., Tuck E., Stubbs V.E., Livesey F.J. (2020). Variable Outcomes in Neural Differentiation of Human PSCs Arise from Intrinsic Differences in Developmental Signaling Pathways. Cell Rep..

[40] Janssen A.F.J., Breusegem S.Y., Larrieu D. (2022). Current Methods and Pipelines for Image-Based Quantitation of Nuclear Shape and Nuclear Envelope Abnormalities. Cells.

[41] Piper M.D.W., Partridge L. (2016). Protocols to Study Aging in Drosophila. Methods Mol. Biol..

