# Supplementary material for: NAT10 inhibition corrects nuclear defects in tau mutant human neurons and extends lifespan in a Drosophila tauopathy model
Source: iScience. 2026 Jul 22;29(8):116861. doi: 10.1016/j.isci.2026.116861 (PMC13426208; doi:10.1016/j.isci.2026.116861)
Supplement: Document S1. Figures S1–S5 [file mmc1.pdf]

iScience, Volume *29*

Supplemental information

NAT10 inhibition corrects nuclear defects in tau mutant human neurons and extends lifespan in a *Drosophila* tauopathy model

Francesco Paonessa, Bernardo Delarue Bizzini, Tom Campbell, Emily Coode, Jonathan Lam, Ravi Solanki, Richard Butler, James Smith, Catherine M. Davidson, Delphine Larrieu, Andrea H. Brand, and Frederick J. Livesey

Figure 3 - complete immunoblot

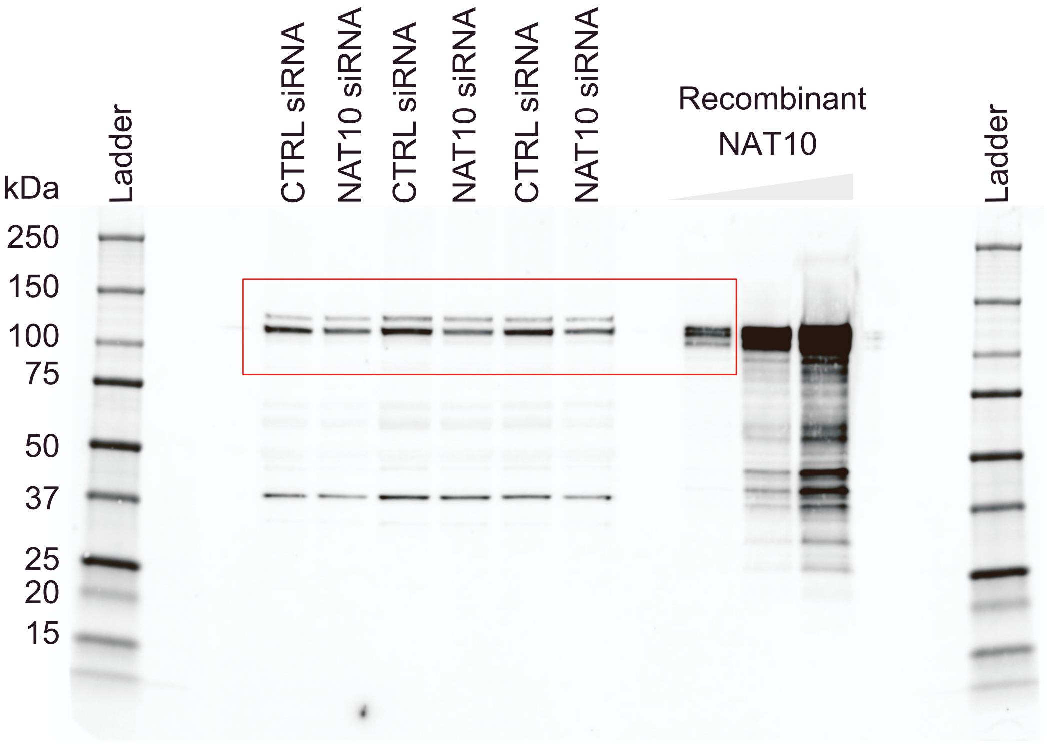

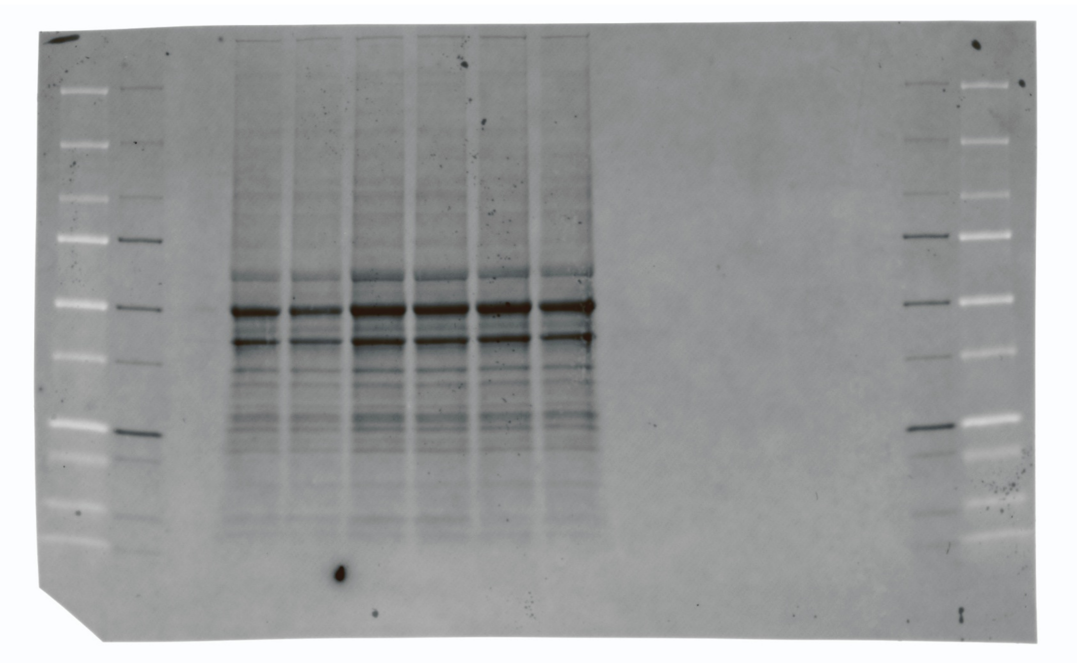

Total protein
(Bio-Rad Stain-free gel; signal from UV-activated trihalo compound)

Ladder = Precision plus Protein All blue Prestained Protein Standards

Area of blot shown in figure 3 is outlined by red box

Figure 6D - complete immunoblot

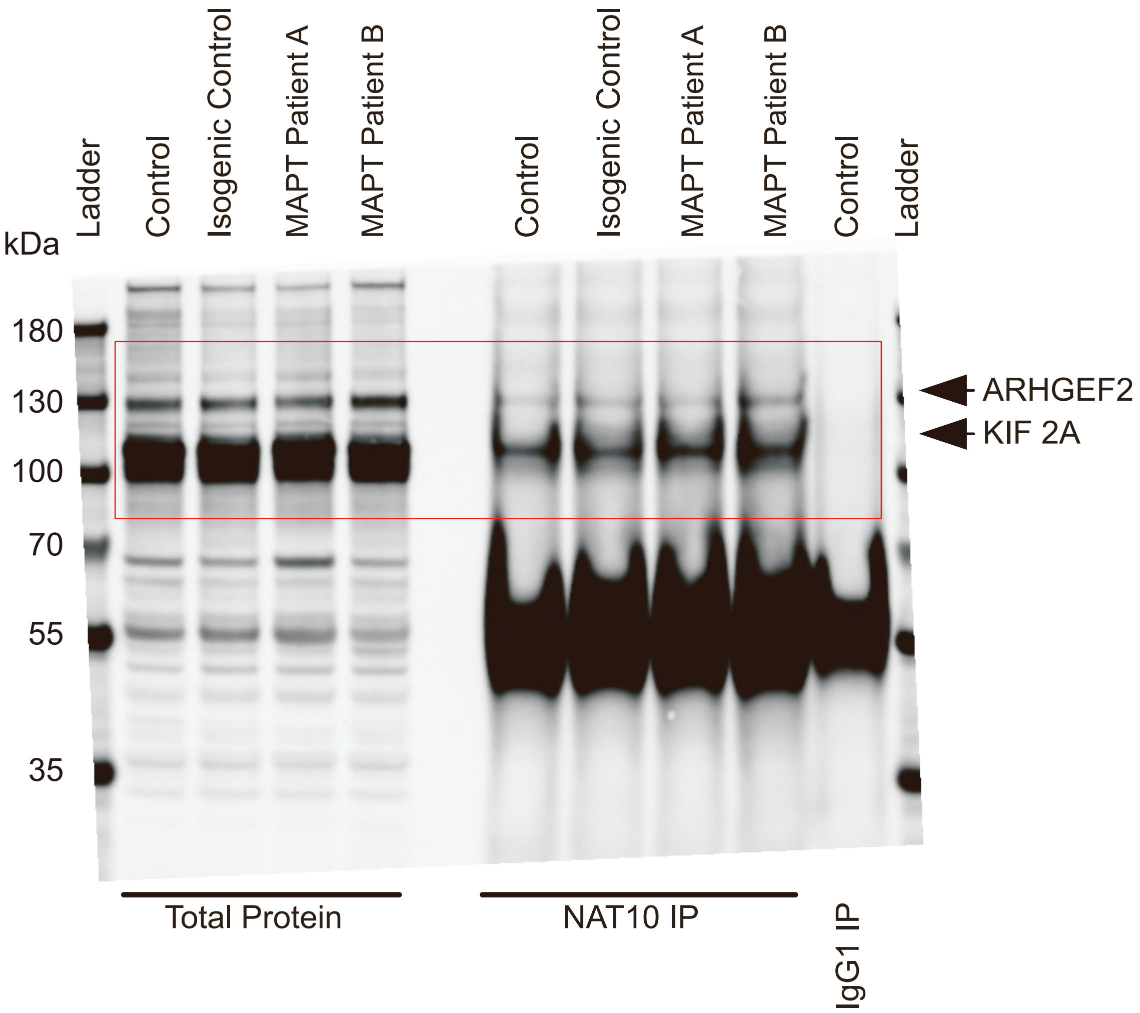

Ladder = Thermo Scientific PageRuler Plus Prestained Protein Ladder

Area of blot shown in figure 6D is outlined by red box

Figure S1 A - complete immunoblots

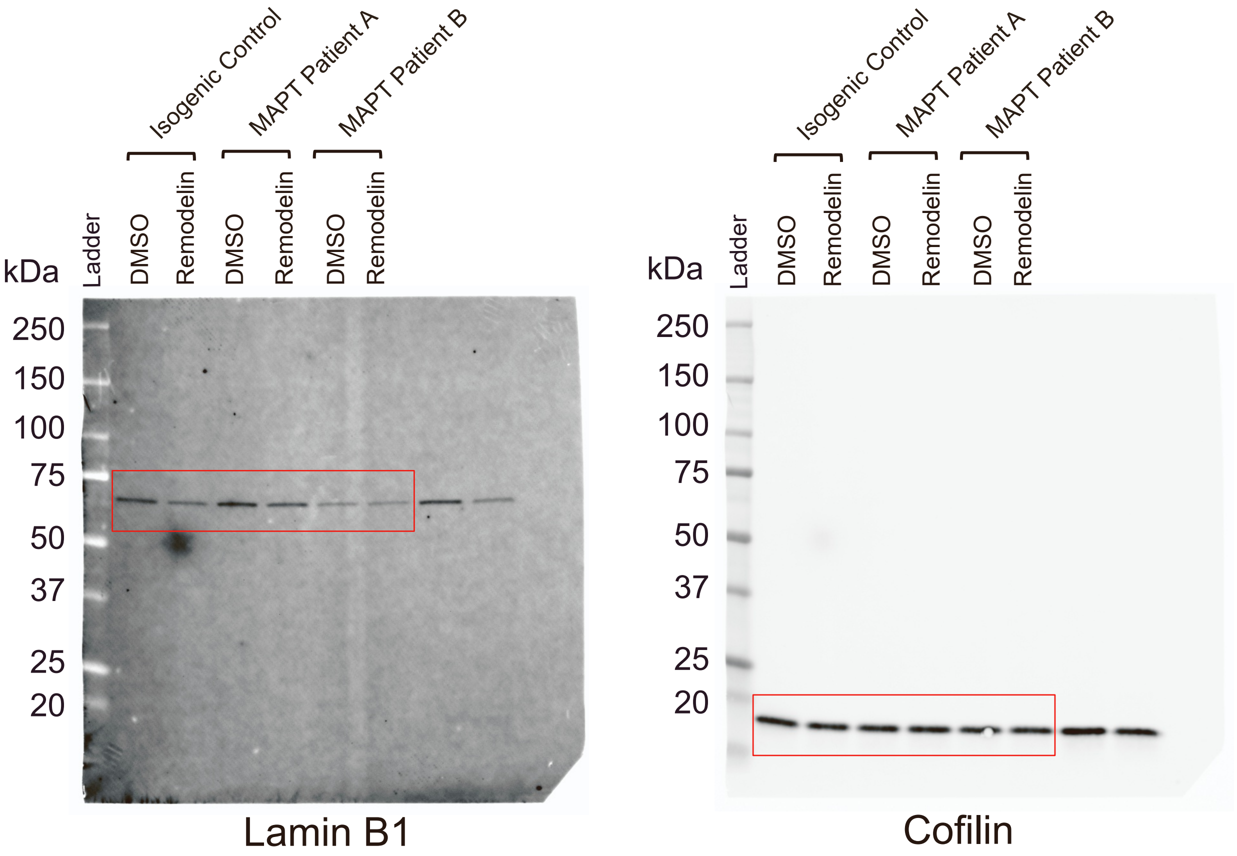

Figure S1 B - complete immunoblot

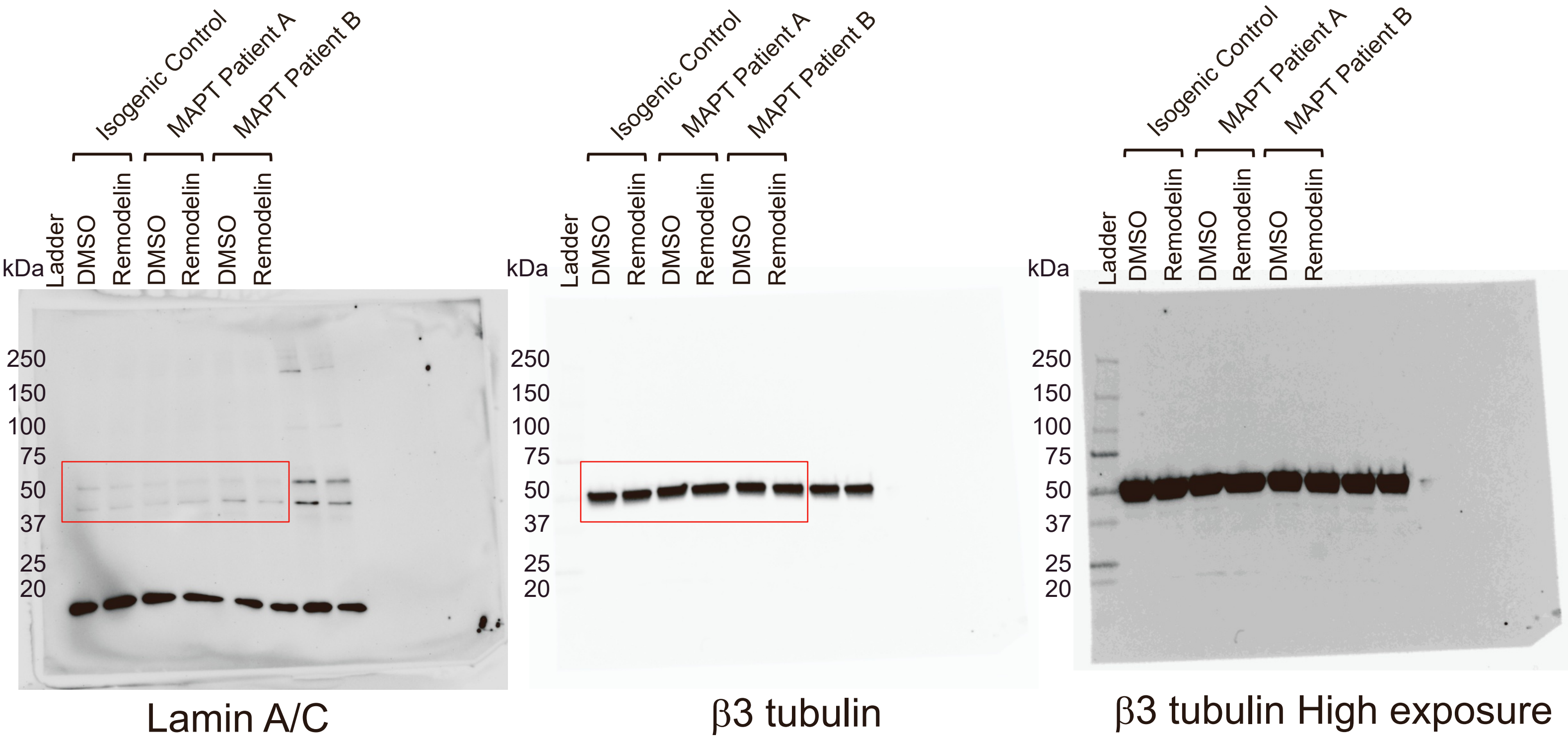

Ladder = Precision plus Protein All blue Prestained Protein Standards

Areas of blots shown in figure S1 are outlined by red box

Figure S4 A (tau p181 and tau p217) - complete immunoblots

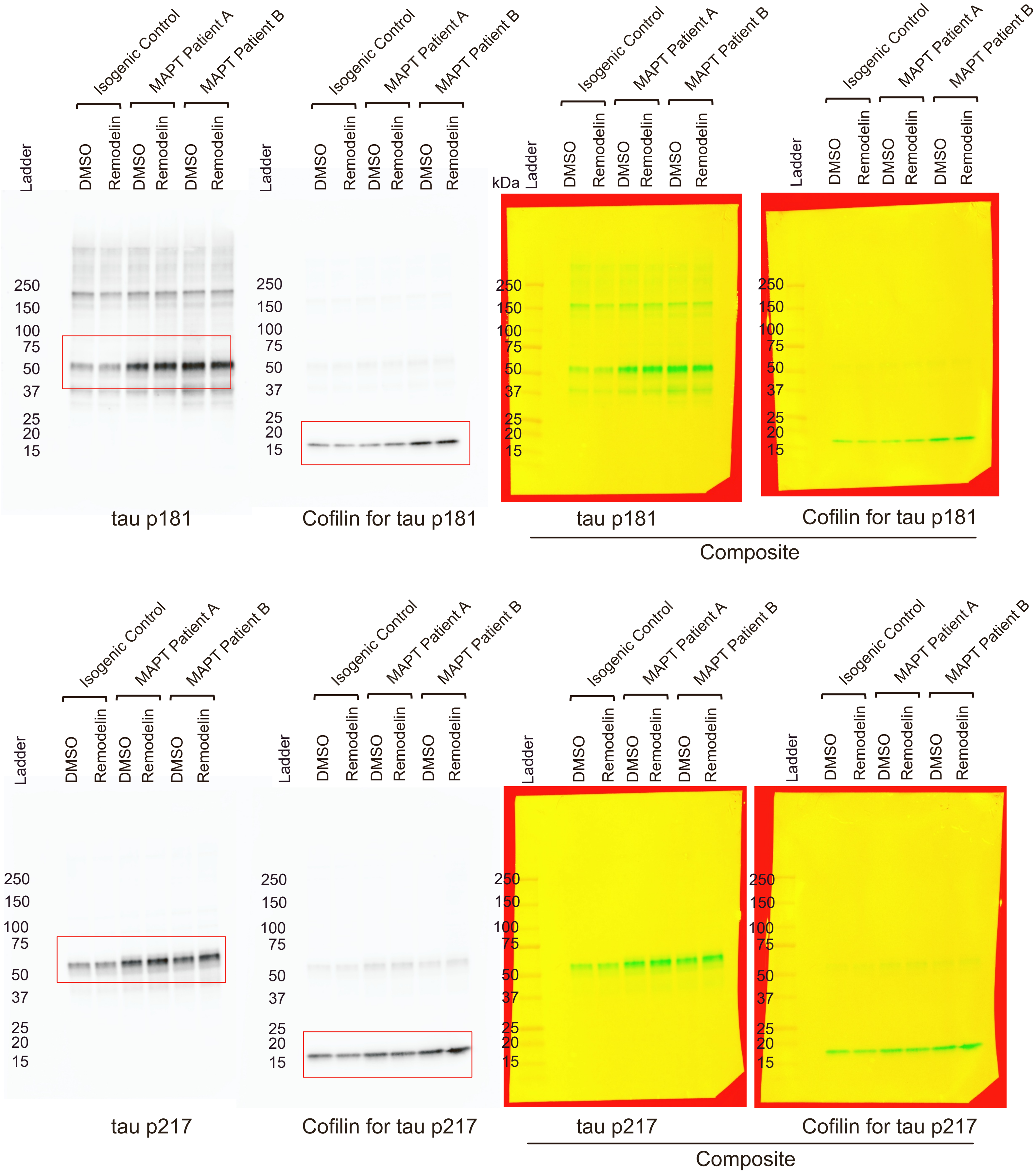

Ladder = Precision plus Protein All blue Prestained Protein Standards

Areas of blots shown in figure S4A are outlined by red box

Composite = pseudocoloured merged images of protein ladder (red) and each individual antibody (green)

Figure S4 A (Total tau and tau AT8) - complete immunoblots

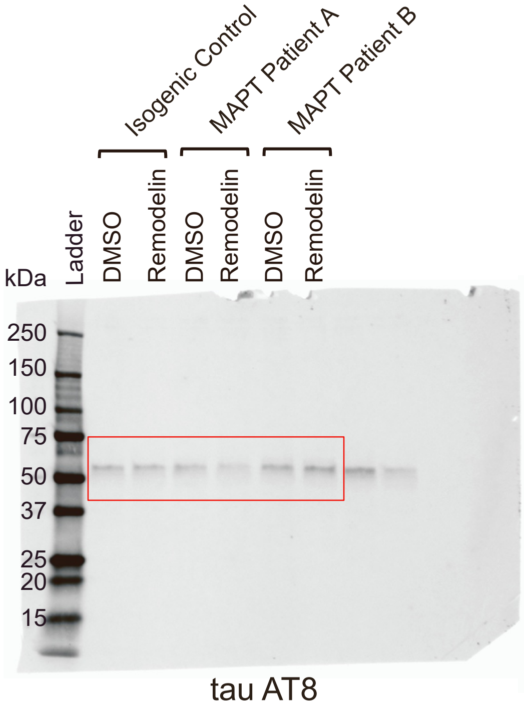

tau AT8

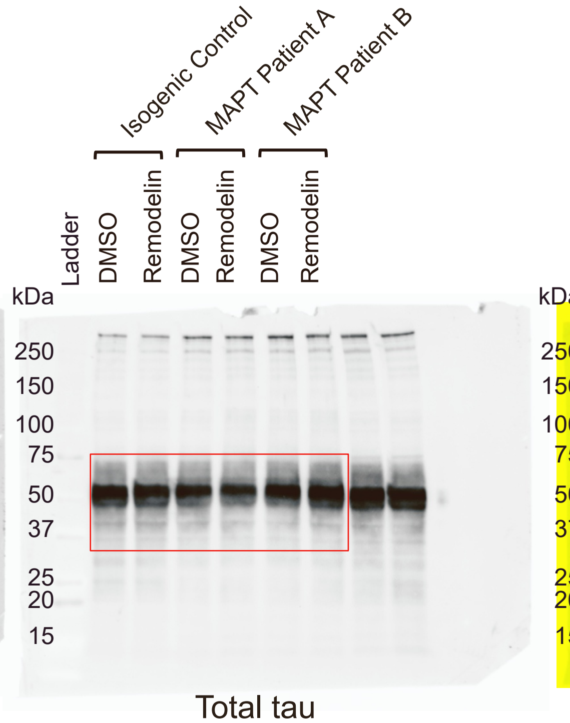

Total tau

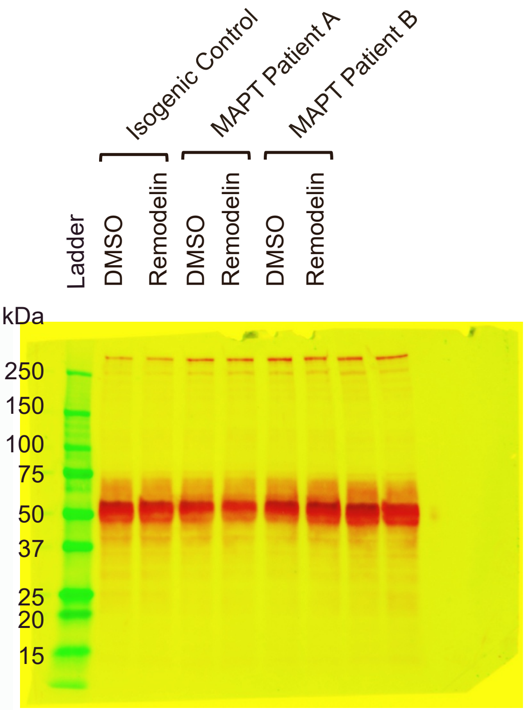

Total tau

Composite

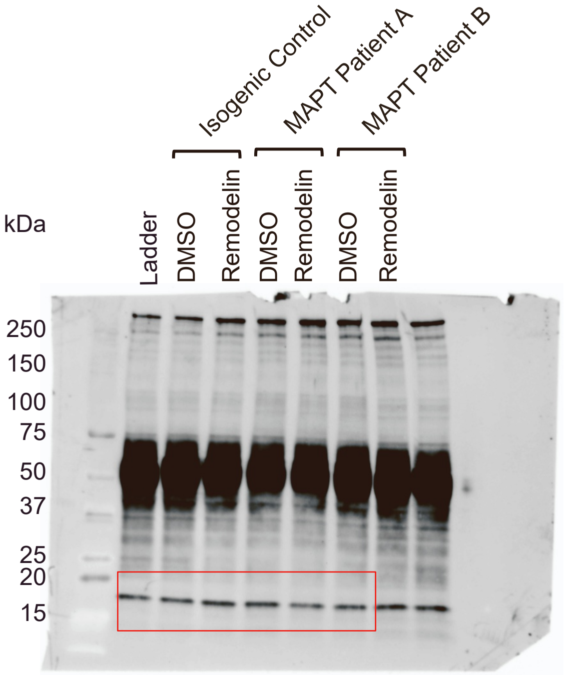

Cofilin for Total tau and tau AT8

Ladder = Precision plus Protein All blue Prestained Protein Standards

Areas of blots shown in figure S4A are outlined by red box

Composite = pseudocoloured merged images of protein ladder (green) and individual antibody (red)

Figure S4 B - complete immunoblots

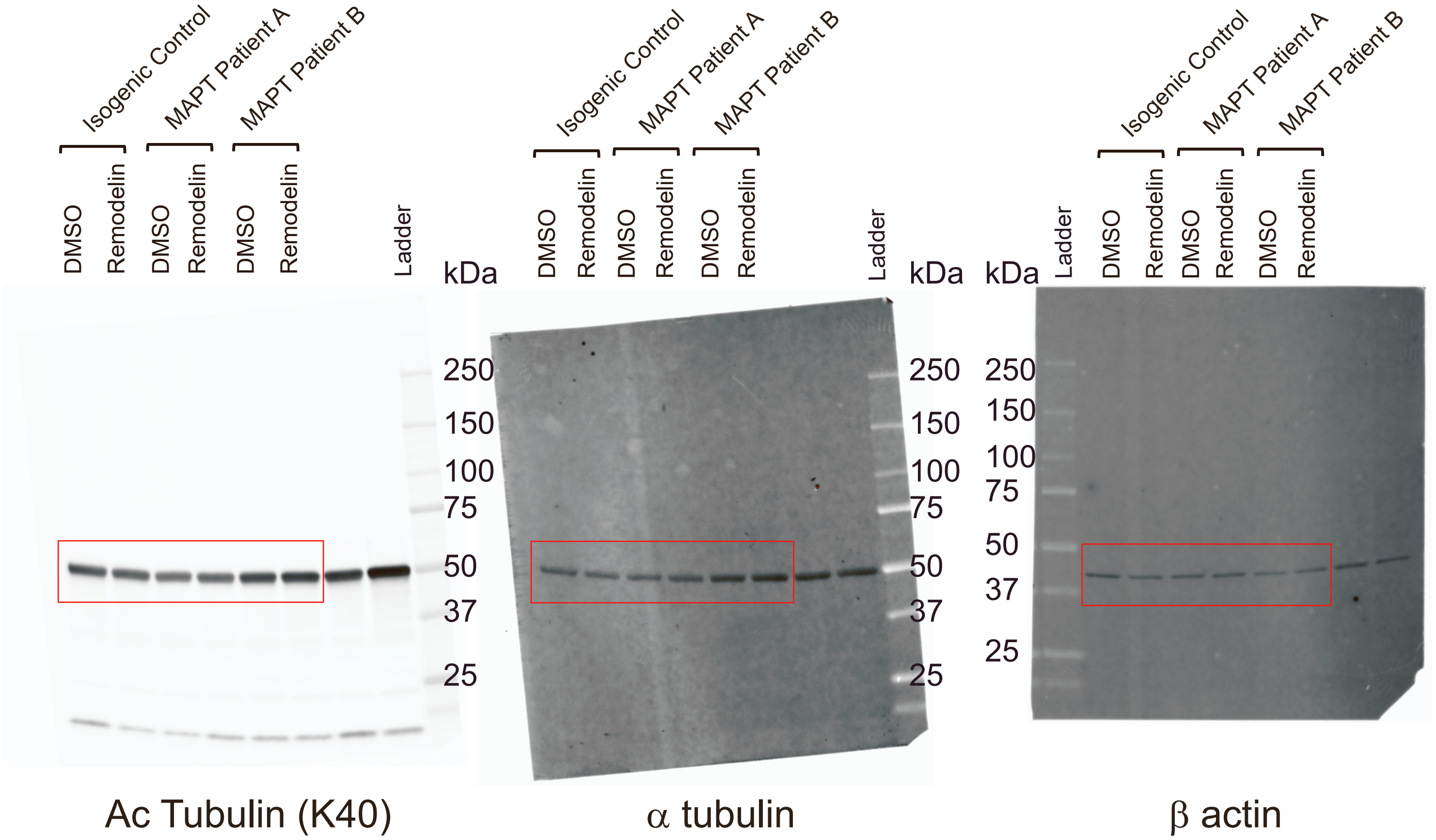

Total protein
(Bio-Rad Stain-free gel; signal from UV-activated trihalo compound)

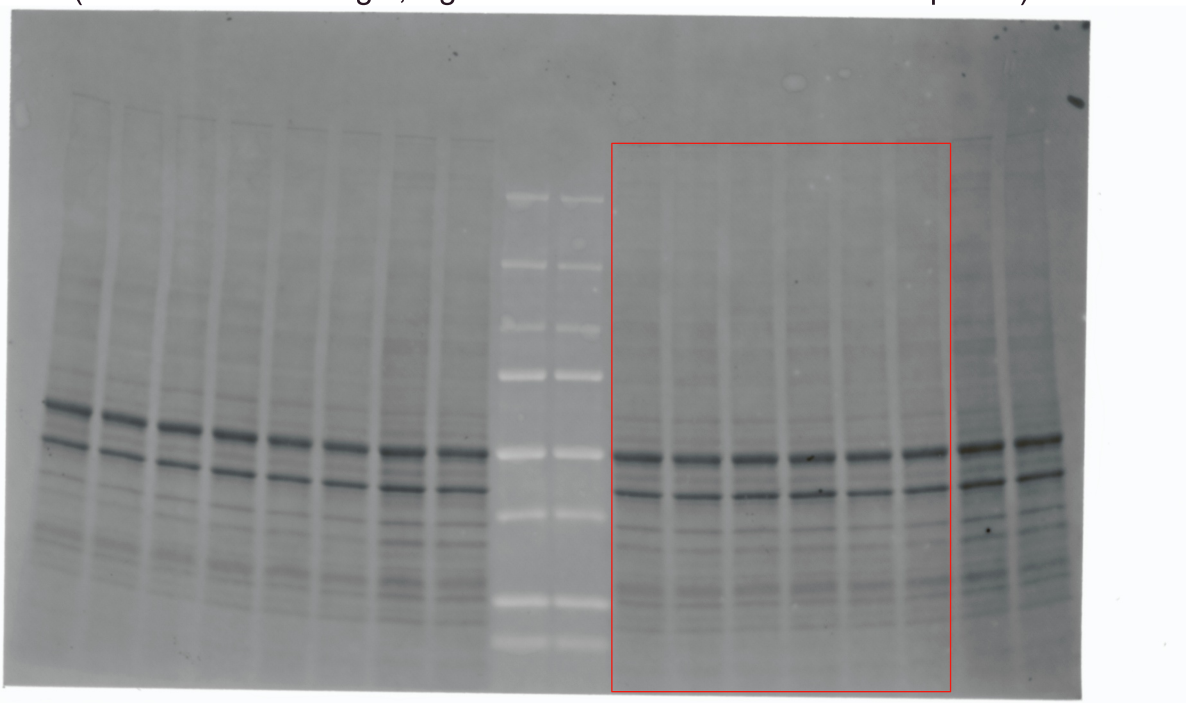

Ac Tubulin (K40) and α tubulin WB β actin WB

Ladder = Precision plus Protein All blue Prestained Protein Standards

Areas of blots shown in figure S4B are outlined by red box
