# Supplementary material for: NAT10 inhibition corrects nuclear defects in tau mutant human neurons and extends lifespan in a Drosophila tauopathy model
Source: iScience. 2026 Jul 22;29(8):116861. doi: 10.1016/j.isci.2026.116861 (PMC13426208; doi:10.1016/j.isci.2026.116861)
Supplement: Document S2. Paonessa uncropped blots [file mmc2.pdf]

Figure S1

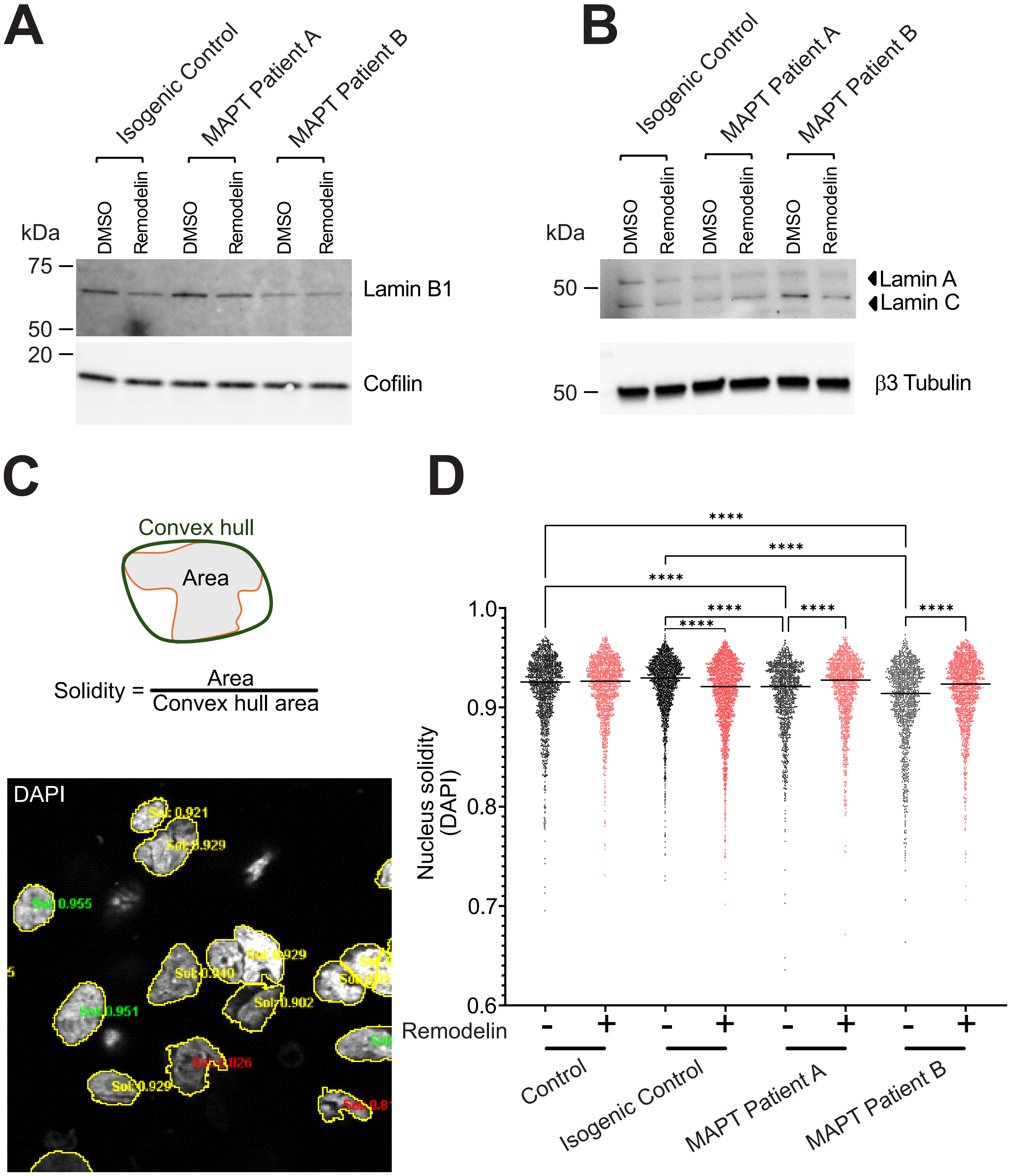
A
Isogenic Control
MAPT Patient A
MAPT Patient B
DMSO
Remodelin
kDa
75
50
20
Lamin B1
Cofilin
B
Lamin A
Lamin C
β3 Tubulin
C
Convex hull
Area
Solidity = Area / Convex hull area
DAPI
D
****
Nucleus solidity (DAPI)
1.0
0.9
0.8
0.7
0.6
Remodelin
-
+
Control
Isogenic Control
MAPT Patient A
MAPT Patient B

Supplementary Figure S1. Lamin expression in human FTD neurons. Related to Figure 1.

A. Lamin B protein expression in isogenic control and FTD neurons exposed to vehicle (DMSO) or Remodelin for 48 hours. Lamin B expression varies and cell lines and with Remodelin treatment. Cofilin expression was compared for reference and as a loading control.

B. LaminA/C protein expression in control and FTD neurons exposed to vehicle (DMSO) or Remodelin for 48 hours, with β3-tubulin as reference control.

C. *Top*, cartoon illustrating nucleus solidity metrics. *Bottom*, representative image from nuclei scored for nucleus solidity (green = high solidity; yellow = intermediate solidity; red = low solidity).

D. FTD-MAPT neurons had a lower nucleus solidity compared with control neurons in Vehicle (-). Nucleus solidity was significantly increased in FTD neurons after treatment with Remodelin (+). As the data were not normally distributed, it was transformed to an approximately normal distribution using an inverse sigmoid transformation. A two-way ANOVA including an interaction term was then performed, followed by Tukey's post-hoc tests (****$p < 0.0001$), data are represented violin plot, the horizontal bar represent the median ; $n > 1000$ nuclei from 3 independent experiments, each dot represent one nucleus).

Figure S2

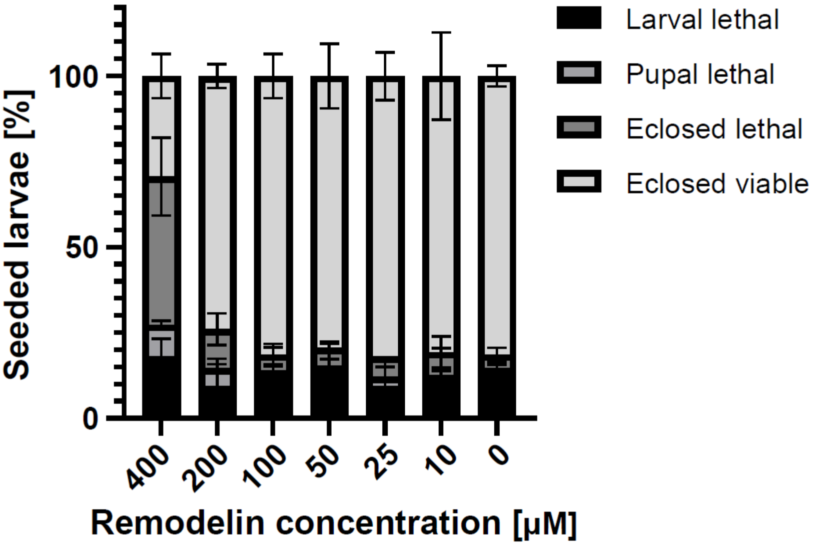

Seeded larvae [%]
100
50
0
400
200
100
50
25
10
0
Remodelin concentration [µM]
Larval lethal
Pupal lethal
Eclosed lethal
Eclosed viable

Supplemenray Figure S2. Dose selection for Remodelin administration to Drosophila. Related to Figure 2.

Drosophila were fed from larval stages on a range of Remodelin concentrations and viability scored at larval, pupal and eclosed stages. 100 μM Remodelin was selected empirically as the highest concentration without toxicity and effects on viability for experiments in Figure 2. Data are represented as mean +/- SD.

Figure S3

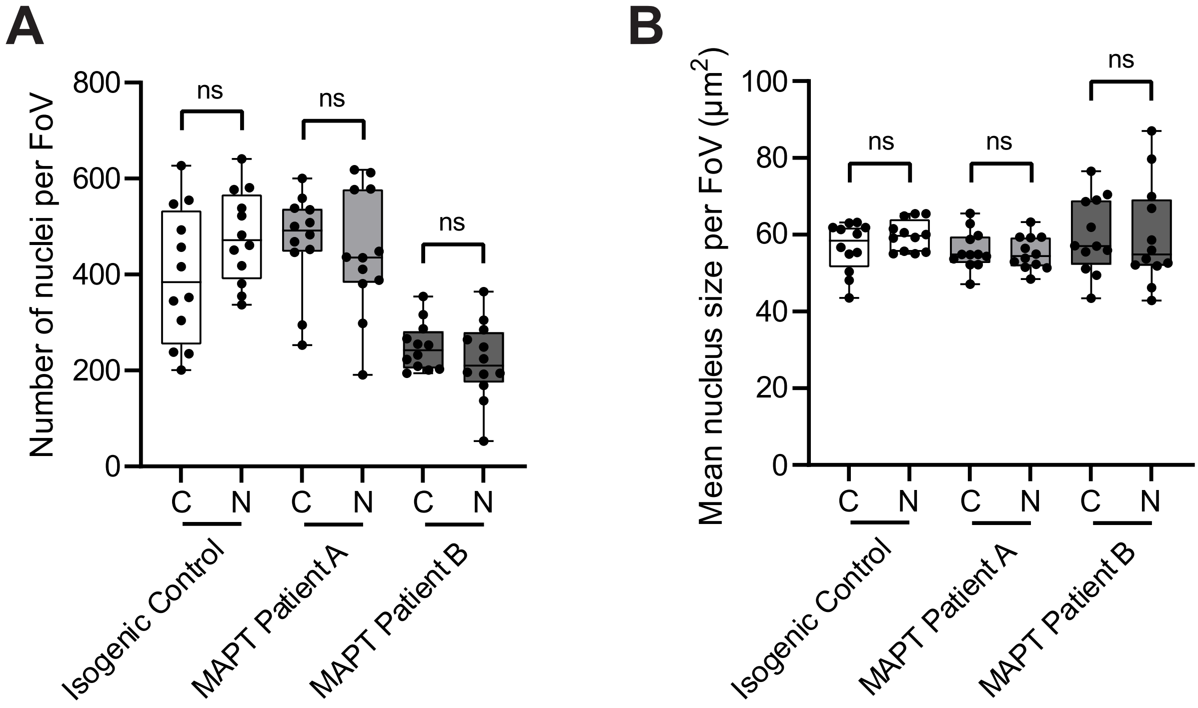
A
Number of nuclei per FoV
0
200
400
600
800
ns
ns
ns
C
N
C
N
C
N
Isogenic Control
MAPT Patient A
MAPT Patient B
B
Mean nucleus size per FoV (µm²)
0
20
40
60
80
100
ns
ns
ns
C
N
C
N
C
N
Isogenic Control
MAPT Patient A
MAPT Patient B

Supplementary Figure S3. NAT10 siRNA knockdown does not alter human neuron viability. Related to Figure 3.

A. For the treatment groups reported in Figure 3, total cell number was determined by counting DAPI-positive objects. No change in total cell number was observed after treatment of neurons with siRNA targeting NAT10 compared to the scramble siRNA control. Significance was determined using unpaired t test with Welch's correction (ns = not significant); data are represented as box-and-whisker plot, boxes represent the median and interquartile range; whiskers indicate minimum and maximum values; each dot represents a field of view.
B. For the treatment groups reported in Figure 3, neuronal viability was also determined by measuring nuclear size, in order to detect the presence of pyknotic cells . No change in nuclear size was observed after treatment of neurons with siRNA targeting NAT10 compared to the scramble siRNA control. Significance was determined using unpaired t test with Welch's correction (ns = not significant); data are represented as box-and-whisker plot, boxes represent the median and interquartile range; whiskers indicate minimum and maximum values; each dot represents a field of view.

Figure S4

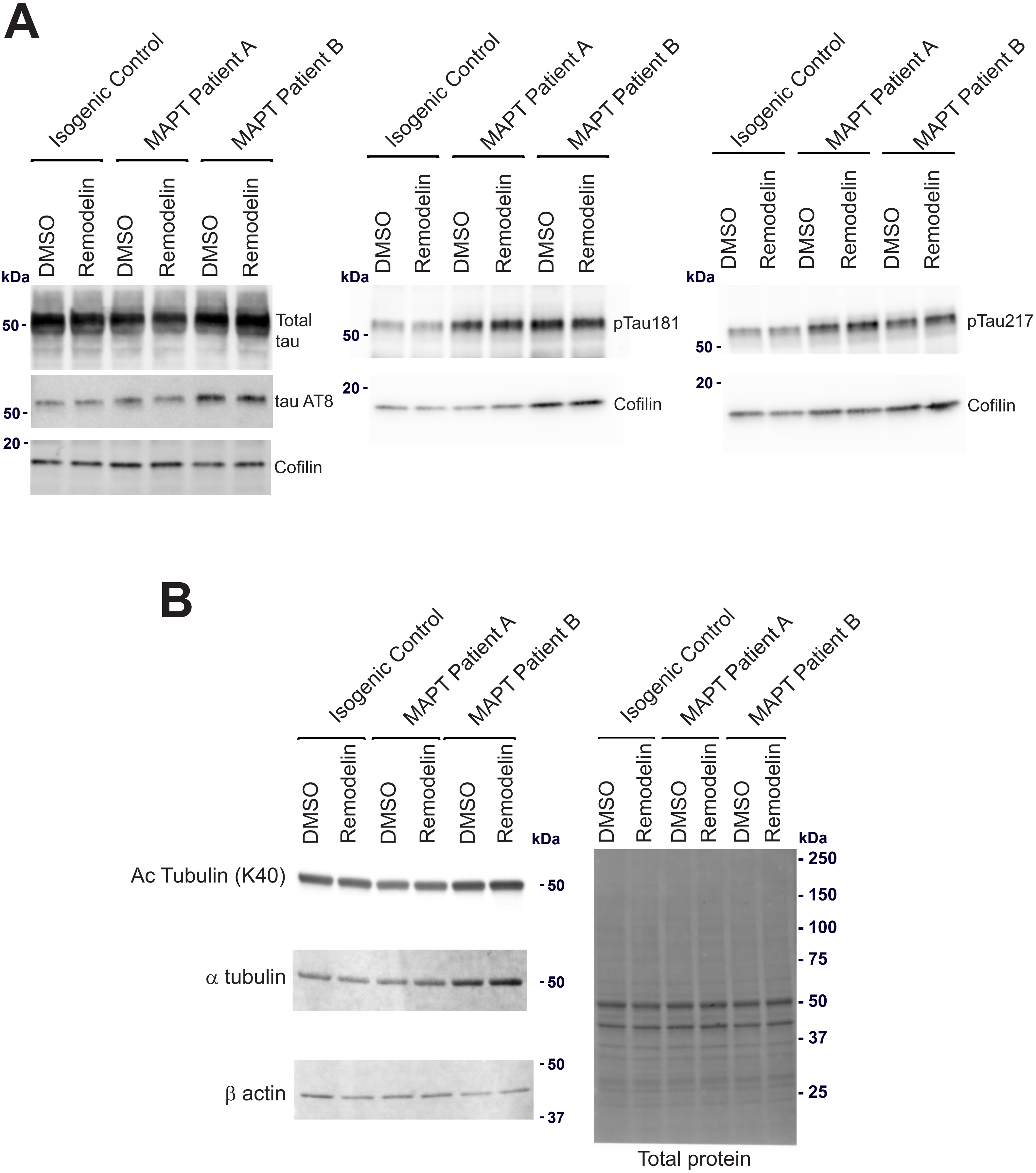
A
Isogenic Control
MAPT Patient A
MAPT Patient B
DMSO
Remodelin
kDa
50
20
Total tau
tau AT8
Cofilin
pTau181
pTau217
B
Ac Tubulin (K40)
α tubulin
β actin
37
250
150
100
75
25
Total protein

Supplementary Figure S4. Effects of Remodelin treatment on cytoskeletal protein levels. Related to Figure 6.

A. No change in total or phosphorylated tau (AT8, tau p181 and tau p217) in isogenic control and FTD neurons treated with Remodelin for 48 hours, compared with vehicle (DMSO). Note that, as previously reported, tau phosphorylation is increased in FTD neurons. Cofilin detection was included for reference and as a loading control.

B. No change in acetylated tubulin (K40), α-tubulin and β-actin in control and FTD neurons treated with Remodelin for 48 hours, compared to vehicle (DMSO). Total protein staining of the same membrane used for tubulin and actin western blotting is shown as a loading control.

Figure S5

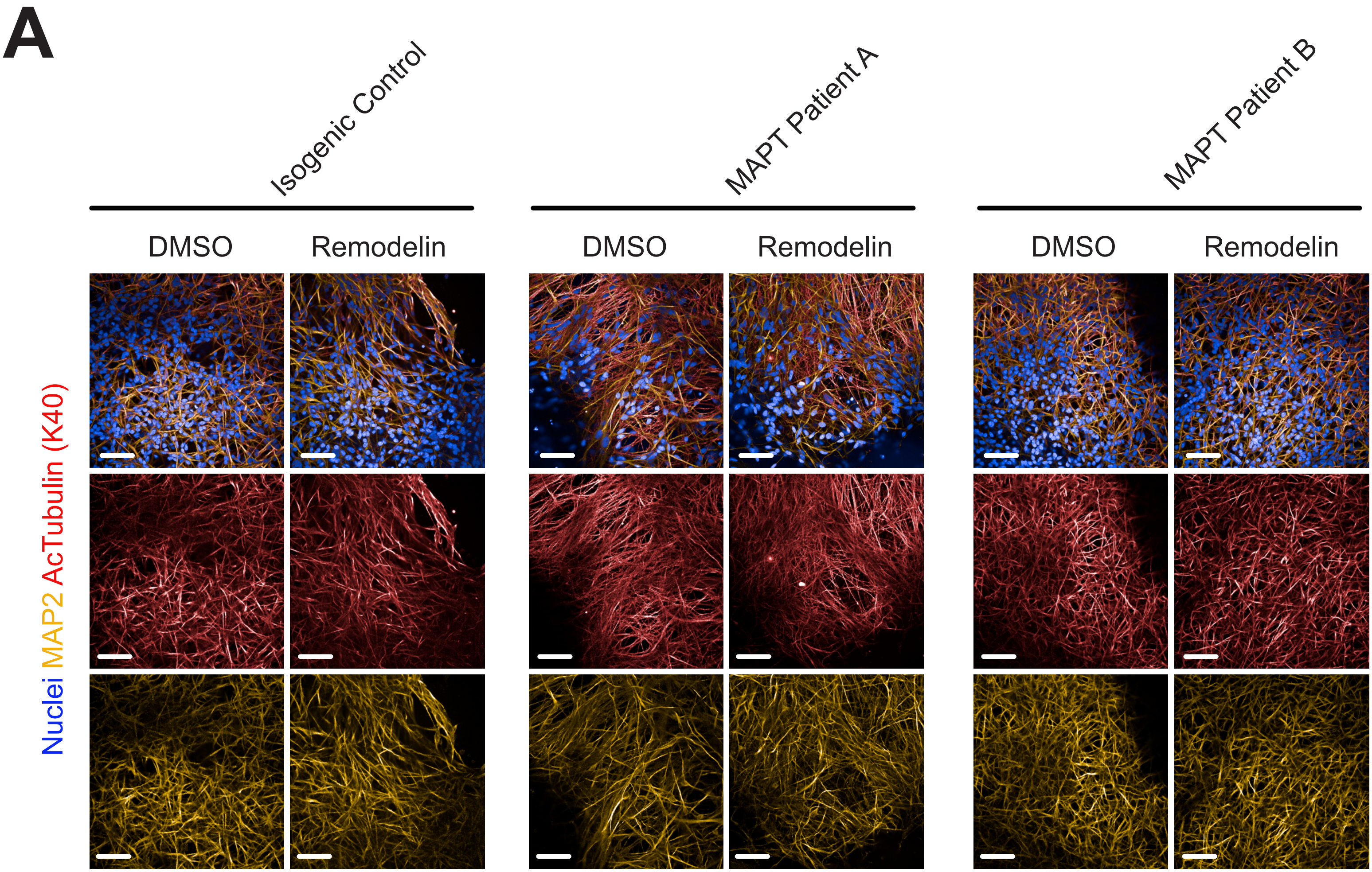
A
Isogenic Control
MAPT Patient A
MAPT Patient B
DMSO
Remodelin
DMSO
Remodelin
DMSO
Remodelin
Nuclei MAP2 AcTubulin (K40)

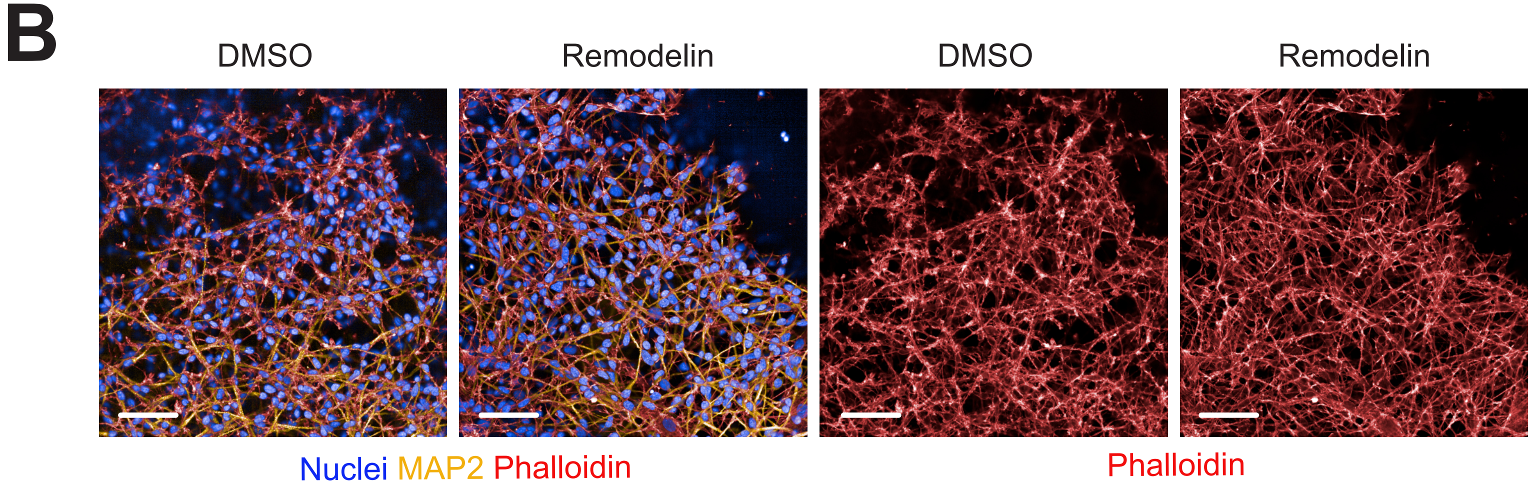
B
DMSO
Remodelin
DMSO
Remodelin
Nuclei MAP2 Phalloidin
Phalloidin

Supplementary Figure S5. Short-term treatment with Remodelin does not have pronounced effects on the organisation of the cytoskeleton. Related to Figure 6.

A. Representative images from isogenic control and FTD-neurons exposed to DMSO or Remodelin for 48 hours and stained for acetylated tubulin (red) and MAP2 (amber). Scale bar = 50 µm.

B. Representative images from isogenic revertant control exposed to DMSO or Remodelin for 48 hours and stained using phalloidin (red) to visualize filamentous actin (F-actin), MAP2 (amber). Scale bar = 50 µm.
